# Clickable Radiocomplexes With Trivalent Radiometals for Cancer Theranostics: *In vitro* and *in vivo* Studies

**DOI:** 10.3389/fmed.2021.647379

**Published:** 2021-06-11

**Authors:** Alice D'Onofrio, Francisco Silva, Lurdes Gano, Urszula Karczmarczyk, Renata Mikołajczak, Piotr Garnuszek, António Paulo

**Affiliations:** ^1^Centro de Ciências e Tecnologias Nucleares, Instituto Superior Técnico, Universidade de Lisboa, Campus Tecnológico e Nuclear, Lisbon, Portugal; ^2^Departamento de Engenharia e Ciências Nucleares, Instituto Superior Técnico, Universidade de Lisboa, Lisbon, Portugal; ^3^National Centre for Nuclear Research, Radioisotope Centre POLATOM, Otwock, Poland

**Keywords:** *in vivo* click-chemistry, radiometals, iEDDA, pre-targeting, theranostics

## Abstract

Pre-targeting approaches based on the inverse-electron-demand Diels-Alder (iEDDA) reaction between strained trans-cyclooctenes (TCO) and electron-deficient tetrazines (Tz) have emerged in recent years as valid alternatives to classic targeted strategies to improve the diagnostic and therapeutic properties of radioactive probes. To explore these pre-targeting strategies based on *in vivo* click chemistry, a small family of clickable chelators was synthesized and radiolabelled with medically relevant trivalent radiometals. The structure of the clickable chelators was diversified to modulate the pharmacokinetics of the resulting [^111^In]In-radiocomplexes, as assessed upon injection in healthy mice. The derivative DOTA-Tz was chosen to pursue the studies upon radiolabelling with ^90^Y, yielding a radiocomplex with high specific activity, high radiochemical yields and suitable *in vitro* stability. The [^90^Y]Y-DOTA-Tz complex was evaluated in a prostate cancer PC3 xenograft by *ex-vivo* biodistribution studies and Cerenkov luminescence imaging (CLI). The results highlighted a quick elimination through the renal system and no relevant accumulation in non-target organs or non-specific tumor uptake. Furthermore, a clickable bombesin antagonist was injected in PC3 tumor-bearing mice followed by the radiocomplex [^90^Y]Y-DOTA-Tz, and the mice imaged by CLI at different post-injection times (p.i.). Analysis of the images 15 min and 1 h p.i. pointed out an encouraging quick tumor uptake with a fast washout, providing a preliminary proof of concept of the usefulness of the designed clickable complexes for pre-targeting strategies. To the best of our knowledge, the use of peptide antagonists for this purpose was not explored before. Further investigations are needed to optimize the pre-targeting approach based on this type of biomolecules and evaluate its eventual advantages.

## Introduction

Radiometals have always played a pivotal role in nuclear medicine and, in recent years, the renewed interest in theranostics further contributed to their spread and popularity (1–3). This broad group of radioisotopes in fact, is extremely diverse and includes alpha, beta and Auger emitters for therapy as well as gamma and positron emitters for SPECT and PET imaging (4, 5). The macrocycle DOTA is considered one of the gold standards in nuclear medicine and in the last 25 years, DOTA-based chelators had a tremendous impact in various medical imaging modalities such as PET, SPECT, MRI and fluorescence imaging (6). Its widespread use is mainly due to its versatility that allows the complexation of different metal ions, but also to its easy functionalization, allowing the most diversified pre-clinical and clinical applications (7–10).

Beside their well-established use in classical targeted radionuclide therapy, radiometal complexes have also been successfully applied to pre-targeting strategies, an approach used in several pre-clinical studies to achieve higher tumor/non-tumor ratio and reduce the overall radiation exposure. Amongst the several pre-targeting systems available, the inverse electron demand Diels-Alder (iEDDA) click reaction between tetrazines (Tz) and trans-cyclooctenes (TCO), emerged because of its extremely fast kinetics and high *in vivo* specificity (11). Due to their high reactivity, Tz and TCO can undergo degradation or isomerization processes *in vivo* leading to the loss of their functionality. However, most of these processes are nowadays known and several derivatives with improved chemical stability have been developed (12, 13). The targeting biomolecule is injected first and, when a satisfactory target accumulation is achieved, is followed by the injection of the radiolabelled small molecule to produce the desired on-site and *in vivo* conjugation (14–17). This approach has proven to be especially useful in radioimmunotherapy (RIT), since the slow pharmacokinetics of antibodies requires several days for their accumulation in the tumor and delivers high radiation doses to healthy tissues (18, 19). Several groups have been reporting on the synthesis of tetrazine containing DOTA-based chelators in the last years upon functionalization with different pegylated linkers and albumin-binding moieties aiming to improve their pharmacokinetic behavior for imaging and therapeutic purposes (20–23). Recently, the theranostic couple ^64^Cu and ^67^Cu (positron and β^−^ emitter, respectively) were used in pre-targeted radioimmunotherapy (PRIT) and demonstrated an excellent correlation between the uptake observed in the PET scans and the dose-dependent therapeutic response (24).

**Figure 1:**
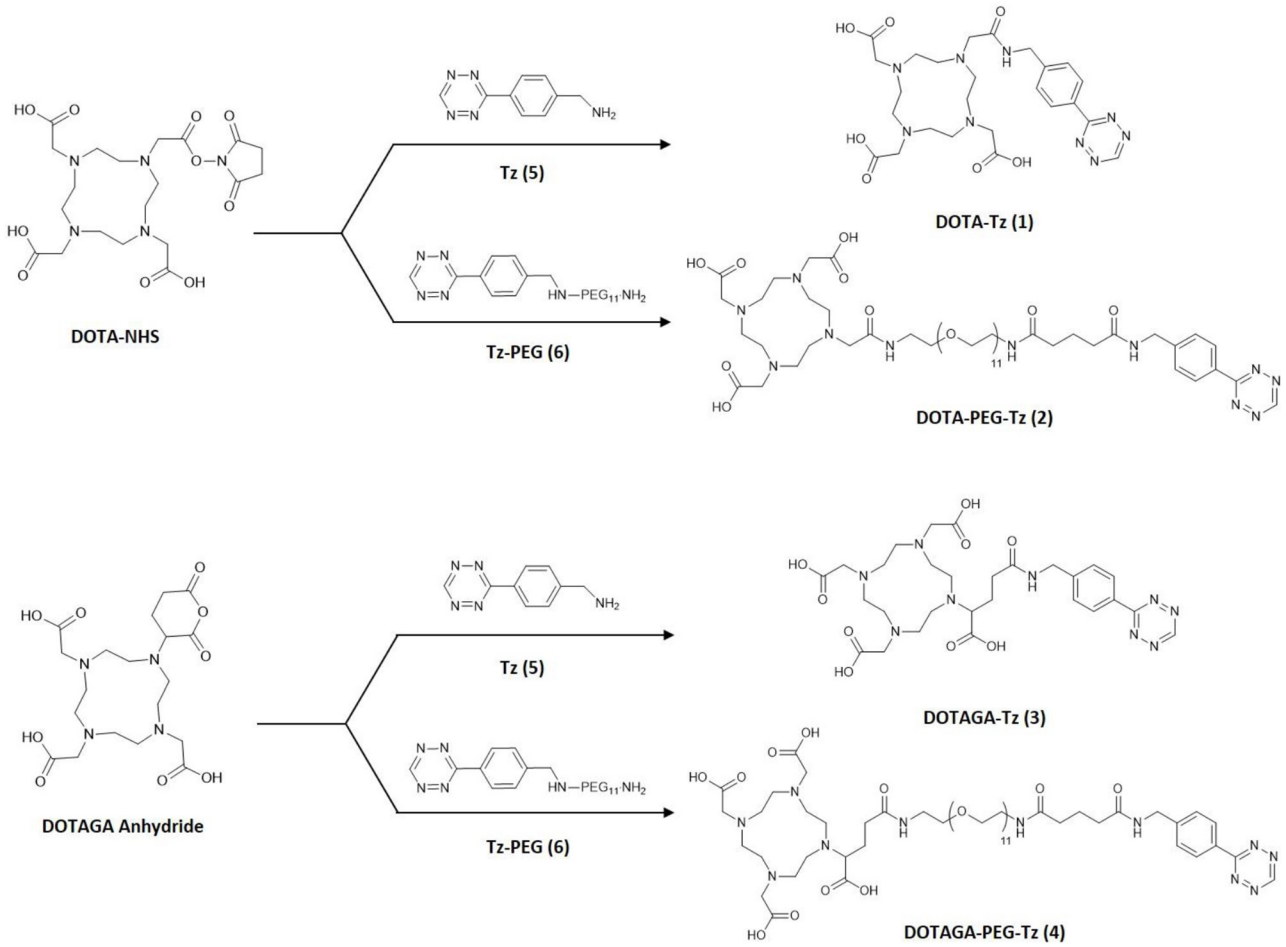
The clickable chelators DOTA-Tz **(1)**, DOTA-PEG-Tz **(2)**, DOTAGA-Tz **(3)** and DOTAGA-PEG-Tz **(4)** synthesized by coupling the commercially available DOTA-NHS and DOTAGA anhydride with the clickable precursors Tz **(5)** and Tz-PEG **(6)**.

In this work, we describe the synthesis and characterization of a small family of DOTA-like clickable chelators for radiometal labeling, shown in Figure 1. These ligands were functionalized with a clickable tetrazine moiety to develop pre-targeting strategies through bio-orthogonal and *in vivo* click chemistry. The choice of the Tz scaffold was based on the easiness of the chemical synthesis and on a compromise between the chemical stability and reactivity for *in vivo* application (12). The chelators, based on DOTA and DOTAGA scaffolds, differ in the number of carboxylate arms and form radiocomplexes with different net charges. Further chemical diversification was obtained by insertion of a polyethylene glycol (PEG) linker in two of the four derivatives. The structural diversity introduced aimed at the modulation of the pharmacokinetics, since DOTA-based radiocomplexes are generally highly hydrophilic and are quickly eliminated from the organism. The overall hydrophilicity and especially the final net charge of the resulting radiocomplexes have been previously reported as crucial parameters to determine their metabolic path through the kidneys or the liver (25–27). The insertion of a pegylated linker, on the other hand, has been associated with a slower blood-clearance and might help to increase the circulation time of the radiocomplexes in the body (21, 28).

The compounds were radiolabelled first with ^111^In, a radionuclide widely used as a surrogate of therapeutic β^−^ emitters, with a relatively similar chemistry and easily available at rather economical prices. The [^111^In]In-radiocomplexes were evaluated for their *in vitro* stability and successively injected in healthy mice to study their pharmacokinetic profile. The best performing clickable chelator, identified based on the studies with ^111^In, was then also radiolabelled with the therapeutic radionuclide ^90^Y. Biodistribution studies and Cerenkov luminescence imaging (CLI) studies were also performed for the [^90^Y]Y-radiocomplex, using a prostate cancer PC3 xenograft, in order to verify possible non-specific retention in the tumors and/or tumor microenvironment, which could compromise its suitability to pre-targeting strategies for cancer theranostics. To have a first insight on its suitability for this purpose, we have evaluated the *in vivo* reaction of this [^90^Y]Y-radiocomplex with a clickable TCO-containing bombesin antagonist (TCO-PEG4-AR) targeted at the gastrin releasing peptide receptor (GRPr) that is overexpressed in PC3 tumors (29).

## Materials and Methods

### Chemical Synthesis

The clickable chelators were obtained starting from the tetrazine containing precursors Tz (5) and Tz-PEG (6). These precursors were successively coupled with the commercially available DOTA-NHS ester and DOTAGA-anhydride, as shown in Figure 1. The couplings were carried out in dry DMF in the presence of NEt_3_ and by stirring the reaction mixture at room temperatures during 3 h to afford the desired final compounds DOTA-Tz (1), DOTAPEG-Tz (2), DOTAGA-Tz (3) and DOTAGAPEG-Tz (4). The chemical synthesis of the derivative DOTAGA-Tz (3) has been previously reported (23). The clickable TCO-containing bombesin antagonist (TCO-PEG4-AR) was performed by coupling the peptide D-Phe-Gln-Trp-Ala-Val-Gly-His-Sta-Leu-NH_2_ (AR) with TCO-PEG4-NHS, in solution and in presence of DIPEA. The chemical synthesis, purification and characterization of the different compounds are detailed in the Supplementary Material.

### Radiosynthesis

The synthesis of the different clickable radiocomplexes is presented below; the synthesis of the radioconjugates [^111^In]In-DOTA-Tz-TCO-PEG4-AR and [^90^Y]Y-DOTA-Tz-TCO-PEG4-AR is described in the Supplementary Material.

#### [^111^In]In-Radiocomplexes

DOTA-Tz, DOTAPEG-Tz, DOTAGA-Tz, and DOTAGAPEG-Tz were dissolved into 0.1 M ammonium acetate buffer at pH 7 to obtain aliquots with a final concentration of 100 μM (~3–5 μL of the ligands in 30–50 μL of buffer). Then, ~15 MBq of [^111^In]InCl_3_ (30–50 μL) were added and the mixtures were incubated at 80°C for 15 min. The radiochemical yield of [^111^In]In-DOTA-PEG-Tz and [^111^In]In-DOTAGA-PEG-Tz was above 95% and the radiocomplexes were used without further purification. The radiocomplexes [^111^In]In-DOTA-Tz and [^111^In]In-DOTAGA-Tz were obtained with an average radiochemical yield (RCY) of 80%. A HPLC purification was performed to isolate the desired radiocomplexes in very high radiochemical purity (>95%) using the Radiometric System I described in the Supplementary Material. The chemical identity of the radiocomplexes was confirmed by HPLC co-injection of the corresponding cold In(III) complexes (see the Supplementary Material) and comparison of the respective UV-chromatograms with the gamma-chromatograms.

**[**^**111**^**In]In-DOTA-Tz** (RCY = 80%, Rt = 12.2 min).

**[**^**111**^**In]In-DOTAGA-Tz** (RCY = 80%, Rt = 14.9 min).

**[**^**111**^**In]In-DOTA-PEG-Tz** (RCY = 95%, Rt = 16.2 min).

**[**^**111**^**In]In-DOTAGA-PEG-Tz** (RCY = 95%, Rt = 16.4 min).

#### [^90^Y]Y-DOTA-Tz

The ^90^Y-labeling of DOTA-Tz was performed after dissolving the compound into 0.1 M ammonium acetate buffer at pH 7 to obtain aliquots with a final concentration of ~400 μM. Then, activities comprised between 15 and 130 MBq of [^90^Y]YCl_3_ were added and the mixtures were incubated at 80°C for 15 min. The radiocomplex [^90^Y]Y-DOTA-Tz was obtained with radiochemical yields above 90% and with specific activity up to 3.8 MBq/nmol. A HPLC purification was performed to isolate the desired radiocomplex in very high radiochemical purity (>95%) using the Radiometric System II described in the Supplementary Material.

**[**^**90**^**Y]Y-DOTA-Tz** (RCY = 90%, Rt = 10.5 min).

### Animal Studies

The *ex-vivo* biodistribution studies performed for the different clickable ^111^In-radiocomplexes, [^90^Y]Y-DOTA-Tz and radiopeptides [^111^In]In-DOTA-Tz-TCO-PEG4-AR and [^90^Y]Y-DOTA-Tz-TCO-PEG4-AR are detailed in the Supplementary Material.

The animal studies were conducted in conformity with the national law and with the EU Guidelines for Animal Care and Ethics in Animal Experimentation. Experimental procedures were carried out in conformity with the National Legislation and the Council Directive of the European Communities on the Protection of Animals Used for Experimental and Other Scientific Purposes (2010/63/UE) and the “ARRIVE guidelines for reporting animal research.” The POLATOM protocol was approved by the Ist Local Animal Ethics Committee in Warsaw (authorization 877/2019, approval date 12 June 2019). Further details on biodistribution studies performed in healthy and in PC3-tumor bearing mice are presented in the Supplementary Material.

### Optical Imaging

The Cerenkov Luminescence Imaging of the prostate cancer PC3 tumor-bearing mice was carried out at different time points (15 min, 1 and 2 h) after intravenous injection of [^90^Y]Y-DOTA-Tz. For the *in vivo* click chemistry studies in the same PC3 xenografts, a bolus of 1 nmol of TCO-PEG4-AR, dissolved in 0.1 mL of saline, was injected in the tail vein, and after 4 h the [^90^Y]Y-DOTA-Tz (0.1 mL, 7.5 μCi) was injected. Thereafter, the CLI imaging of the PC3 tumor-bearing mice was carried out at different time points (15 min, 1 h). The images were obtained using PhotonIMAGER™ (BioSpace Lab). The optical imaging system was based on intensified CCD camera (25 mm), which had a minimum detectable radiance of 37 photons/s/sr/cm^2^, minimum image pixel resolution of 2.5 μm and temporal resolution of 23 ms.

## Results

### Synthesis of the Clickable Chelators and Complexation With ^nat^In

The four final clickable ligands DOTA-Tz (**1**), DOTA-PEG-Tz (**2**), DOTAGA-Tz (**3**), and DOTAGA-PEG-Tz (**4**) were analyzed by HPLC and characterized by ESI-MS (Supplementary Table 1). The final coupling reactions showed poor reaction yields, especially for the derivatives DOTA-Tz (**1**) and DOTAGA-Tz (**3**). Nonetheless, this drawback might be eventually improved by further optimizing the reactions, namely by increasing the reaction time and using different bases and/or solvents. The complexation of the four compounds with the In^3+^ ion using ^nat^In, has been detailed in the Supplementary Material. The resulting complexes were characterized by common analytical techniques (Supplementary Table 1) and were successively used as surrogates to identify the corresponding radiocomplexes on the basis of their chromatographic profiles and retention times, expected to be identical.

### Clickable [^111^In]In-Radiocomplexes

The radiolabelling of DOTA-Tz and DOTAGA-Tz was optimized by dissolving the compounds into ammonium acetate buffer 0.1 M and pH 7, to afford the corresponding [^111^In]In-DOTA-Tz and [^111^In]In-DOTAGA-Tz radiocomplexes in 80% average yields. HPLC purification allowed to achieve a radiochemical purity above 95% (Supplementary Table 2). The radiochemical yield of the radiocomplexes [^111^In]In-DOTA-PEG-Tz and [^111^In]In-DOTAGA-PEG-Tz was above 95% and the radiocomplexes were used in further studies without additional purification. The chemical identity of the radiocomplexes was ascertained by co-injection in the HPLC of the cold ^nat^In congeners and comparison between the respective UV-chromatograms and radiochromatograms (Supplementary Figure 1).

**Figure 2:**
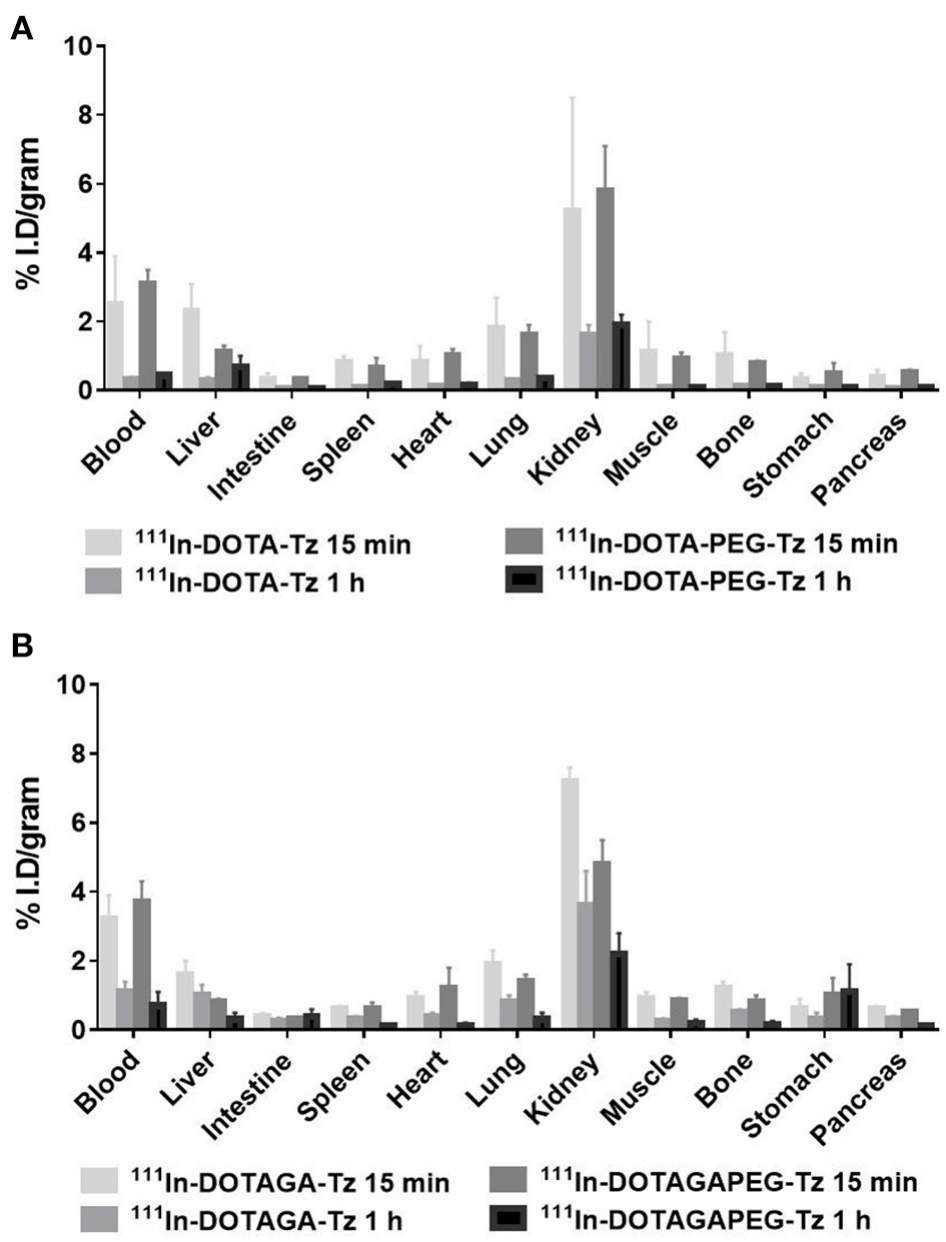
Biodistribution data (% I.D./g ± SD) for [^111^In]In-DOTA-Tz and [^111^In]In-DOTA-PEG-Tz in the top image **(A)** and [^111^In]In-DOTAGA-Tz and [^111^In]In-DOTAGA-PEG-Tz in the bottom image **(B)**. The studies were performed in healthy mice 15 min and 1 h p.i. (*n* = 3–5).

We performed a biodistribution study of the four [^111^In]In-radiocomplexes in healthy mice to determine the clearance rate from systemic blood circulation and the tissue/organ distribution over time. Results were expressed as percentage of the injected activity per gram of tissue (% I.D./g), as shown in Figure 2. The time points chosen, tailored according to the expected short biological half-lives of the [^111^In]In-radiocomplexes, included the early time point of 15 min and a later 1 h time point, when over 80% of the radioactivity injected was eliminated. This result on one side allows to reduce the exposure of potential patients to systemic non-targeted radiation, but on the other side limits the time available for the clickable radiocomplexes to find their chemical counterpart at the tumor site and to react. The urinary tract was identified as the main excretory pathway since the highest % I.D./g values were found in the kidneys for all the radiocomplexes, associated to rapid radioactivity excretion both at 15 min and 1 h p.i. At 15 min the kidney uptake was 5.2 ± 3.3 and 5.8 ± 1.3% I.D./g for [^111^In]In-DOTA-Tz and [^111^In]In-DOTA-PEG-Tz, respectively and 7.2 ± 0.4 and 4.8 ± 0.7% I.D./g for [^111^In]In-DOTAGA-Tz and [^111^In]In-DOTAGA-PEG-Tz, respectively. At the later time point of 1 h, kidney uptake values of 1.6 ± 0.3 and 1.9 ± 0.3% I.D./g were observed for [^111^In]In-DOTA-Tz and [^111^In]In-DOTA-PEG-Tz, respectively, while for the derivatives [^111^In]In-DOTAGA-Tz and [^111^In]In-DOTAGA-PEG-Tz the values observed were 3.6 ± 1 and 2.2 ± 0.6% I.D./g, respectively (Supplementary Table 3).

Low liver uptake was observed for all radiocomplexes, with the highest value of 2.3 ± 0.8 and 1.6 ± 0.4 of the % I.D./g measured at 15 min p.i., for [^111^In]In-DOTA-Tz and [^111^In]In-DOTAGA-Tz, respectively. Lower liver uptake values, around 1% I.D./g, were detected for the pegylated radiocomplexes. The compounds [^111^In]In-DOTA-Tz and [^111^In]In-DOTA-PEG-Tz had very similar excretion values 1 h after injection, above 90% of the injected dose. At the same time point, the compounds [^111^In]In-DOTAGA-Tz and [^111^In]In-DOTAGA-PEG-Tz showed a slower excretion rate, ranging between 81.5 and 87.1% of the injected dose. The non-pegylated derivative [^111^In]In-DOTAGA-Tz was the one showing the lowest value of excretion among the four radiocomplexes. The net charge of the DOTAGA-based complexes seemed to have a higher influence than the pegylated linker in extending the half-life of the radiocomplexes (Supplementary Table 3). The same trend was reported previously for an enlarged family of related macrocyclic derivatives of the NOTA-Tz and NODA-Tz types, labeled with ^18^F and ^68^Ga and carrying different PEGylated linkers. For these clickable tracers, a faster clearance from blood circulation was also observed for those with highest overall net charge (30). Nevertheless, when looking at the effect of the PEG11 linker exclusively on the blood half-life, the pegylated derivatives seem to have slightly higher blood activities compared to the non-pegylated radiocomplexes. At the time point of 15 min, values of 2.5 ± 1.4 vs. 3.1 ± 0.4 of the % I.D./g were observed for [^111^In]In-DOTA-Tz and [^111^In]In-DOTA-PEG-Tz, respectively, and of 3.2 ± 0.7 vs. 3.7 ± 0.6 of the % I.D./g for [^111^In]In-DOTAGA-Tz and [^111^In]In-DOTAGA-PEG-Tz, respectively. At 1 h p.i. the same trend was observed for the DOTA-based radiocomplex with uptake values of 0.3 ± 0.07 vs. 0.44 ± 0.07 of the % I.D./g for the derivative [^111^In]In-DOTA-Tz and [^111^In]In-DOTA-PEG-Tz, respectively. Contrary, the trend was reversed for the DOTAGA-based derivatives with values of 1.1 ± 0.3 vs. 0.7 ± 0.4 of the % I.D./g for [^111^In]In-DOTAGA-Tz and [^111^In]In-DOTAGA-PEG-Tz, respectively.

To assess the *in vivo* stability of the radiocomplexes, samples of blood and urine were collected from the mice injected with the compounds and analyzed by HPLC. The activity in the blood 1 h p.i. was too low to be detected in the HPLC system, as the majority of the radiocomplexes were quickly eliminated. The analysis of the urine samples corresponding to the radiocomplexes [^111^In]In-DOTAGA-Tz and [^111^In]In-DOTA-PEG-Tz 1 h p.i. indicated an overall low degree of metabolization, being observed additional radioactive peaks with similar retention time and likely corresponding to catabolites with higher hydrophilicity. Minor peaks corresponding to free ^111^In were also observed (see Supplementary Figure 2). By contrast, the derivative [^111^In]In-DOTA-Tz was apparently eliminated without undergoing any metabolization or degradation, an encouraging result for future *in vivo* applications. These results allow to speculate that this radiocomplex is likely to circulate in the bloodstream mostly intact.

Given the limited effect of the pegylated linker on the plasmatic half-life, we focused our successive *in vitro* stability studies and lipophilicity measurements on the two non-pegylated derivatives [^111^In]In-DOTA-Tz and [^111^In]In-DOTAGA-Tz. The lipophilicity was evaluated by determination of their partition coefficient log Po/w using the shake-flask method. Both clickable radiocomplexes demonstrated to be quite hydrophilic with the −1 charged radiocomplex, [^111^In]In-DOTAGA-Tz, being the most hydrophilic, with an absolute coefficient value almost double than the one of [^111^In]In-DOTA-Tz (log Po/w of −3.44 ± 0.29 and −1.60 ± 0.13, respectively). The *in vitro* stability was assessed by incubation at 37°C in cell culture media (CCM) and in human serum (HS) followed by HPLC analysis of each solution at selected time points (1, 2, 4, and 24 h). The two radiocomplexes showed very good stability in CCM during the first 4 h of incubation, but after 24 h high level of degradation of 70% was found (Supplementary Figure 3). In HS the stability was very good up to 2 h of incubation, but after 4 h of incubation, the degradation started to be quite important with 50 and 30% of the compound degraded for [^111^In]In-DOTA-Tz and [^111^In]In-DOTAGA-Tz, respectively. At 24 h of incubation the radiocomplexes were almost completely degraded. Both radiocomplexes were also incubated in PBS during 24 h at 37°C and showed very good stability (>85%). Taken together, excellent stability of the two [^111^In]In-radiocomplexes was observed both in CCM and HS at 37°C for incubation times up to 2 h. Since the radiocomplexes have very short biological half-lives and are eliminated rather quickly, these results led us to anticipate that *in vivo* the major part of the radiocomplexes should reach their TCO counterparts intact, and undergo the click reaction.

Considering the results obtained in the radiolabelling, *in vivo* and *in vitro* studies of the four [^111^In]In-radiocomplexes, we decided to focus the following studies with the therapeutic β^−^ emitter ^90^Y on the clickable chelator DOTA-Tz. This choice was motivated by the higher *in vivo* stability and by the easy separation of the radiolabelled [^111^In]In-DOTA-Tz derivative from the unreacted chelator. Given a future *in vivo* application of such clickable chelator, this advantage might allow to achieve radiocomplexes with higher specific activity reducing the competition risk for the *in vivo* click reaction with the TCO counterpart (31).

### [^90^Y]Y-DOTA-Tz

#### Radiolabelling Studies

The radiolabelling with ^90^Y was performed following the same procedure previously optimized for ^111^In, using a buffer of 0.1 M ammonium acetate at pH 7 and heating the mixture at 80°C for 15 min. The DOTA-Tz chelator was added to a final concentration of 400–500 μM to yield [^90^Y]Y-DOTA-Tz with specific activities up to 3.3 MBq/nmol and radiochemical yields above 90% (Supplementary Figure 4A). The radiocomplex [^90^Y]Y-DOTA-Tz was synthesized with ~130 MBq of [^90^Y]YCl_3_ and obtained in very high specific activity, after separation from the non-labeled DOTA-Tz chelator. The radiocomplex was recovered into phosphate buffer at pH 8 to neutralize the residual TFA from the HPLC mobile phase before its use in future biological studies. However, after 2 h at room temperature, the injection of the radiocomplex in the HPLC revealed a high degradation of the compound, most likely due to radiolysis (Supplementary Figure 4B). In order to stabilize the [^90^Y]Y-DOTA-Tz preparation, a suitable ratio between the volume of phosphate buffer solution at pH 8 to neutralize the TFA from the HPLC purification and ascorbic acid as a radiolytic stabilizer had to be optimized. In initial experiments, the use of ascorbic acid as a buffer in the radiolabelling procedure led to the degradation of the tetrazine moiety. Similar effect was seen when the ascorbic acid solution (50 mg/mL) was added to the solution after labeling. After several attempts, the final ratio of 3 μL of ascorbic acid (50 mg/mL) and 147 μL of phosphate buffer, pH 8 allowed to stabilize the final [^90^Y]Y-DOTA-Tz preparation (Supplementary Figure 5A). The preparation was successively analyzed by HPLC and showed excellent *in vitro* stability up to 20 h after the radiolabelling (Supplementary Figures 5B,C).

#### *In vivo* Evaluation of [^90^Y]Y-DOTA-Tz in Nude Mice With PC3 Xenografts

**Figure 3:**
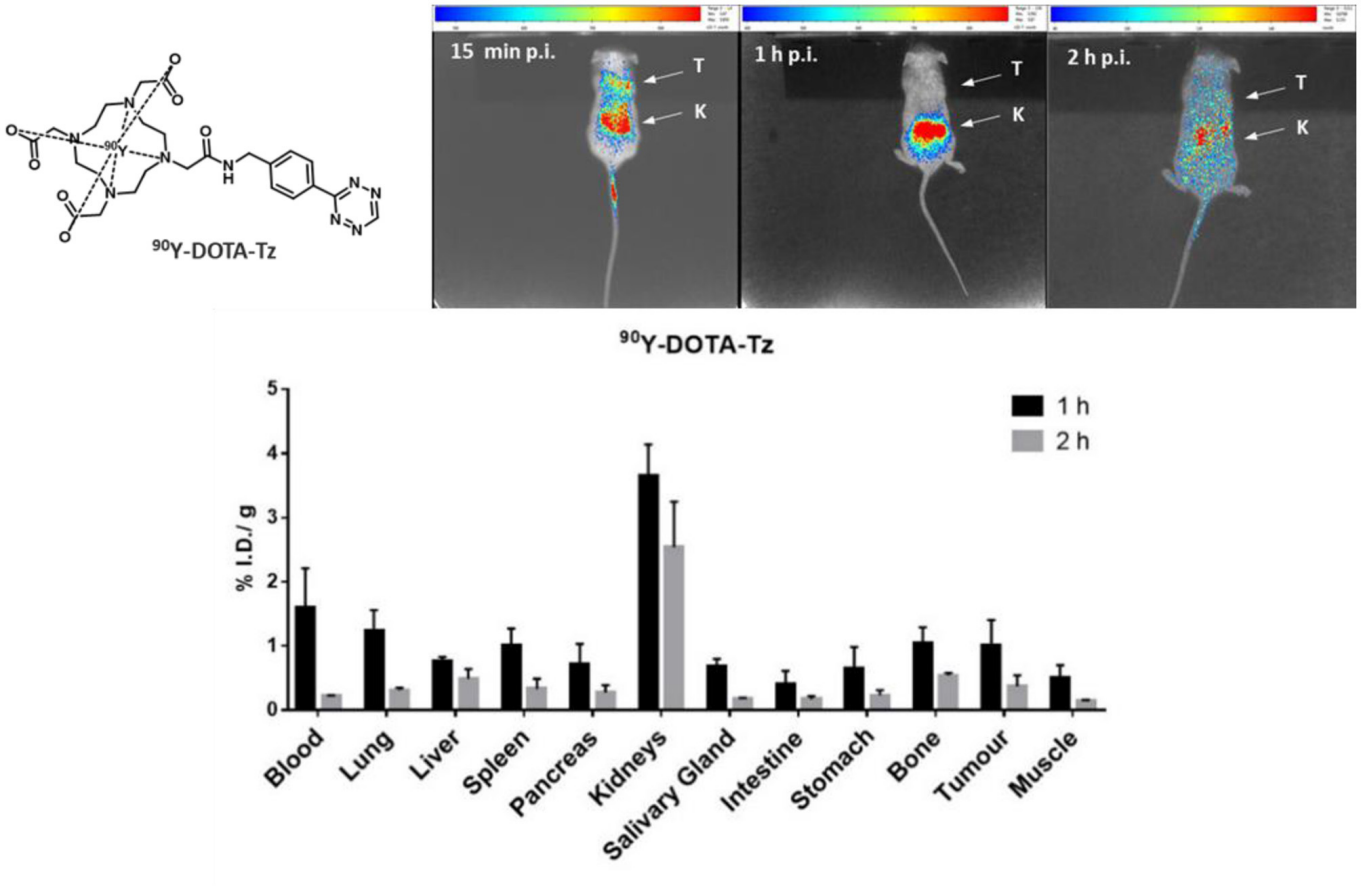
Cerenkov luminescence images in the top image at different time points (15 min, 1 and 2 h) of PC3 tumor-bearing mice administered with [^90^Y]Y-DOTA-Tz [White arrows indicate the tumor (T) and kidneys (K)]. In the bottom image, the biodistribution data for [^90^Y]Y-DOTA-Tz in PC3 xenografts bearing mice, expressed as % I.D./g of organ (mean ± S.D., *n* = 4).

Currently, we are investigating the application of click chemistry using biomolecules that target prostate cancer. Therefore, since a non-specific tumor uptake might interfere with the specificity of the pre-targeting approach, we have performed *in vivo* studies of [^90^Y]Y-DOTA-Tz in mice bearing PC3 xenografts to assess the possible non-specific uptake of the clickable radiocomplex in the tumor and in the tumor microenvironment. The highly energetic β^−^ emission of the radionuclide ^90^Y (maximum particle energy of 2.28 MeV, average energy of 0.94 MeV) allowed to follow the biodistribution of the [^90^Y]Y-DOTA-Tz radiocomplex through CLI imaging. This type of optical imaging offers a high spatial resolution, and despite a limited penetration depth allows to image ^90^Y, which is not easily feasible using PET or SPECT imaging (32). The biodistribution of the [^90^Y]Y-DOTA-Tz radiocomplex was studied in subcutaneous PC3 xenografts in mice and images were acquired at 15 min, 1 and 2 h post-injection (Figure 3). The *ex vivo* biodistribution highlighted very low uptake values in all organs with the highest uptake of 3.6 and 2.5% I.D./g measured in the kidneys at 1 and 2 h p.i. The plasma half-life of [^90^Y]Y-DOTA-Tz was also very short, with uptake values of 1.59 and 0.22% I.D./g in the blood at 1 and 2 h post-injection (Supplementary Table 4). At the selected time points the majority of the compound was retrieved in the urine, with 88 and 96% of the I.D. excreted 1 and 2 h post-injection, respectively.

**Figure 4:**
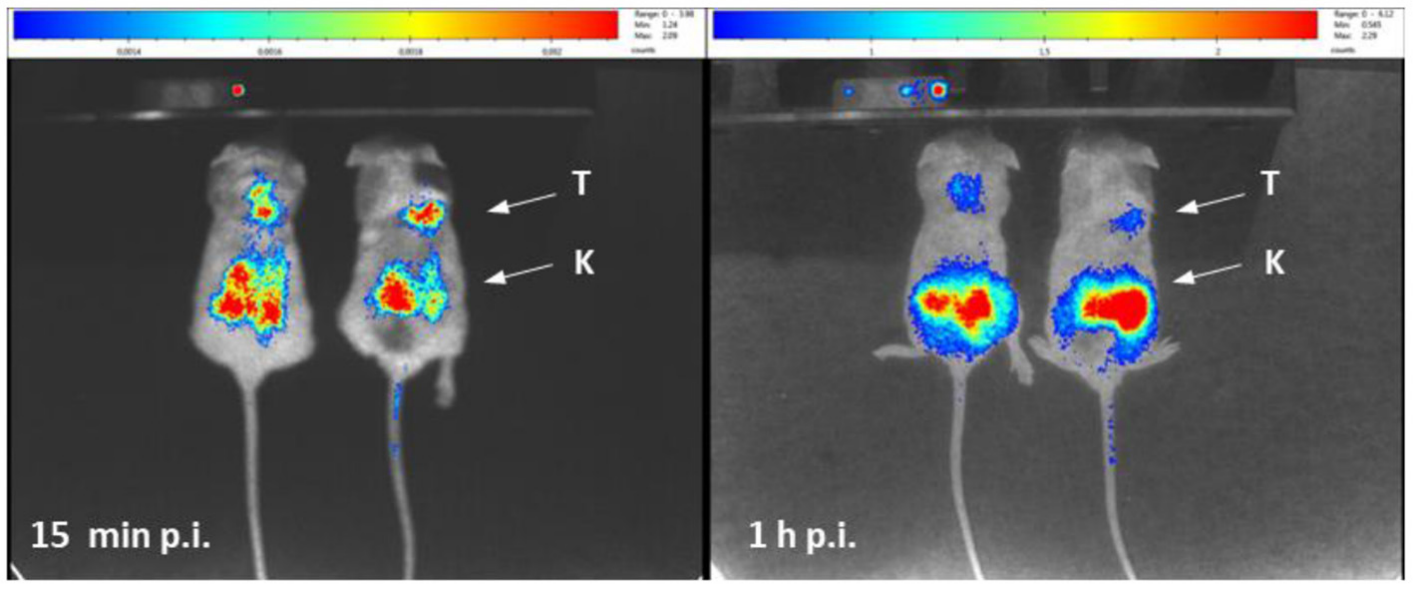
Cherenkov luminescence images at different time points (15 min, 1 h) of prostate cancer PC3 tumor-bearing mice administered first with AR-PEG4-TCO (1 nmol) and then, after 4 h, with [^90^Y]Y-DOTA-Tz.

Searching for a preliminary proof of concept of the intended pre-targeting approach, we synthesized a clickable TCO-containing bombesin antagonist (TCO-PEG4-AR) based on the potent GRPr antagonist AR reported in the literature, as described in the Supplementary Material (29). We hypothesized that the antagonist, when bound to the surface of human PC3 cells, would be available for the *in vivo* click reaction with the Tz-containing radiocomplex. As a control, the biodistribution data of the preformed [^90^Y]Y-DOTA-Tz-TCO-PEG4-AR in PC3-xenografts bearing mice at 15 min and 1 h p.i. are available in Supplementary Table 5. For the pre-targeting approach, the tumor-bearing mice were injected with 1 nmol of the clickable bombesin antagonist and, after 4 h, we injected the radiocomplex [^90^Y]Y-DOTA-Tz. Cerenkov imaging studies were performed 15 min and 1 h after the injection of the radiocomplex, and the analysis of the images at 15 min p.i. seem to indicate some tumor uptake. Overtime, a large part of the activity seemed to be washed away from the tumor site with the majority of the activity localized in the kidneys at 1 after the injection (see Figure 4), but some activity remained in the tumor. In parallel, pre-targeting experiments based on *ex-vivo* biodistribution studies were performed in the same way for the congener [^111^In]In-DOTA-Tz, and using the same animal model. At 1 h p.i., the data from these pre-targeting experiments with ^111^In show an enhanced uptake in the tumor (0.78% I.D./g) when compared with non-target tissues, with the exception of the kidneys (1.5% I.D./g) that are involved in the excretion of the hydrophilic ^111^In-DOTA-Tz clickable complex (Supplementary Table 6). Moreover, these *in vivo* click chemistry results showed relatively favorable tumor/muscle and tumor/blood ratios of 5.2 and 2.8, respectively. Altogether, these results pinpoint the feasibility of the *in vivo* click reaction between [^90^Y]Y-DOTA-Tz and the pre-incubated conjugate TCO-PEG4-AR at the surface of cells overexpressing GRPr. Further and more comprehensive studies are necessary to confirm the viability of this approach and to verify if it could optimize the biodistribution profile of pre-assembled GRPr antagonists radiolabelled through the classical approach.

## Discussion and Conclusions

In this work the chemical synthesis and the analytical characterization of a small family of clickable chelators were presented. The synthetic strategy involved the preparation of two different tetrazine-containing precursors Tz (**5**) and Tz-PEG (**6**) bearing a terminal amino and carboxylate group, respectively, and with the derivative (**6**) additionally functionalized with a pegylated linker. These precursors were used for the coupling with the activated derivatives DOTA-NHS and DOTAGA-anhydride to afford the four clickable chelators DOTA-Tz (**1**), DOTA-PEG-Tz (**2**), DOTAGA-Tz (**3**) and DOTAGA-PEG-Tz (**4**). The four chelators were used to obtain clickable [^111^In]In-radiocomplexes for their *in vivo* evaluation in healthy mice. All radiocomplexes were quickly excreted, demonstrating that their use might contribute to reduce non-targeted radiation dose to the patients. Encouragingly, only minor metabolization processes of the radiocomplexes were revealed by the analysis of urine after 1 h p.i. pointing out that clickable radiocomplexes retain sufficient *in vivo* stability for the intended pre-targeting strategy, particularly in the case of DOTA-Tz (**1**). These results were corroborated by *in vitro* stability studies performed in cell culture media and human serum, where stabilities higher than 90% were observed up to 2 h of incubation at 37°C. Based on the convenient possibility to easily remove the free ligand during HPLC purification, combined with the highest *in vivo* stability of its ^111^In complex, the clickable chelator DOTA-Tz (**1**) was chosen to extend the radiochemical studies to ^90^Y.

The *in vivo* behavior of [^90^Y]Y-DOTA-Tz was evaluated in a prostate cancer PC3 xenograft model by *ex-vivo* biodistribution studies and Cerenkov luminescence imaging. Similarly, the small and hydrophilic radiotracer was quickly eliminated, as predicted by the previous studies with the [^111^In]In-radiocomplexes, and displays a negligible uptake (0.37% I.D./g at 2 h p.i.) in the tumor. The *ex vivo* biodistribution pointed out a short plasma half-life and low uptake values in all organs with excretion occurring mainly via the renal system. Two hours p.i., the majority of the compound was retrieved in the urine, with 96% of the I.D. excreted. Nonetheless, a preliminary proof of concept of the intended pre-targeting approach was obtained by injecting a clickable bombesin antagonist in PC3 tumor-bearing mice followed by the radiocomplex [^90^Y]Y-DOTA-Tz. The analysis of CLI images at 15 min p.i. pointed out a quick tumor uptake but also a fast tumor washout, with the majority of the activity localized in the kidneys at 1 h after the injection. In the present work, we have focused on Cerenkov Luminescence Imaging due to the availability of this modality at our facilities and relevance of ^90^Y for radionuclide therapy. However, in the near future, we intend to extend the study to tomographic nuclear imaging modalities with higher spatial resolution, namely microSPECT imaging using ^111^In.

To the best of our knowledge, the use of TCO derivatives of peptide antagonists for *in vivo* click chemistry is unprecedented. Nevertheless, we are aware that our preliminary results require further extensive investigations aiming to confirm and optimize the pre-targeting approach toward antagonists and to evaluate eventual advantages over classic targeted approach. This should include further control experiments to definitively exclude the possibility that the tumor accumulation involves the clickable [^90^Y]Y-DOTA-Tz and [^111^In]In-DOTA-Tz complexes by themselves, and not necessarily the reaction with the clickable antagonist exposed at the tumor cells surface.

## Data Availability Statement

The original contributions presented in the study are included in the article/Supplementary Material, further inquiries can be directed to the corresponding author/s.

## Ethics Statement

The animal study was reviewed and approved by Ist Local Animal Ethics Committee in Warsaw (authorization 877/2019, approval date 12 June 2019).

## Author Contributions

AP, AD'O, and RM: conceptualization. AD'O: chemical synthesis. AD'O, FS, and PG: radiolabelling. LG and UK: animal studies. AD'O and AP: writing—original draft preparation. AP and RM: funding acquisition. All authors writing—review and editing, have read, and agreed to the published version of the manuscript.

## Conflict of Interest

The authors declare that the research was conducted in the absence of any commercial or financial relationships that could be construed as a potential conflict of interest.
