# Supplementary material for: Clickable Radiocomplexes With Trivalent Radiometals for Cancer Theranostics: In vitro and in vivo Studies
Source: Front Med (Lausanne). 2021 Jun 11;8:647379. doi: 10.3389/fmed.2021.647379 (PMC8225959; doi:10.3389/fmed.2021.647379)
Supplement: Supplementary file 1 [file Data_Sheet_1.pdf]

Supplementary Material

**1**	**General Information**	**2**
**2**	**Nuclear Magnetic Resonance Spectroscopy (NMR)**	**2**
**3**	**Mass Spectrometry: Electrospray Ionization**	**2**
**4**	**High Performance Liquid Chromatography (HPLC)**	**2**
**5**	**Radio-HPLC**	**3**
**6**	**Instant Thin Layer Chromatography (ITLC)**	**3**
**7**	**Chemical Synthesis**	**3**
**8**	**^{nat}In-complexes**	**8**
**9**	**In vivo Studies**	**8**
**10**	**Stability Studies**	**10**
**11**	**Lipophilicity Determination**	**10**
**12**	**Schemes**	**11**
**13**	**Tables**	**12**
**14**	**Figures**	**17**
**15**	**References**	**21**

1 General Information

Handling of air- and/or moisture-sensitive compounds was performed in an inert atmosphere, using vacuum lines and Schlenk techniques. All commercially acquired chemical reagents and solvents were pro-analysis quality, and were used without any additional purification. Solvents were dried and distilled in accordance to literature.(1) All chemicals were purchased from Sigma-Aldrich and used as received, unless otherwise specified. DOTA- NHS and DOTAGA anhydride were purchased from Chematech (France). $InCl_3$ (anhydrous 99%) was acquired from Alfa Aesar (Germany). $^{111}InCl_3$ (370 MBq/mL in HCl) was obtained from Mallinckrodt Medical B.V. (Netherlands). $^{177}LuCl_3$ and $^{90}YCl_3$ were produced and gently provided by the Radioisotope Centre POLATOM, National Centre for Nuclear Research in Otwock, Poland.

2 Nuclear Magnetic Resonance Spectroscopy (NMR)

^{1}H- and ^{13}C-NMR spectra were recorded on Bruker Avance III 400 MHz or 300 MHz spectrometers (IST, Lisbon, Portugal). The chemical shifts are given in ppm and referenced to the residual solvent resonances relative to tetramethylsilane ($SiMe_4$). Coupling constants (J) are given in Hz.

3 Mass Spectrometry: Electrospray Ionization

Mass spectra were acquired in an electrospray ionization/quadrupole ion trap (ESI/QITMS) Bruker HCT mass spectrometer. Samples were diluted in CH_3CN and injected at a flow rate of 150 µL.h^{-1}.

4 High Performance Liquid Chromatography (HPLC)

The HPLC analyses were performed using HPLC grade solvents and bi-distilled H_2O filtered through 0.22 µm Millipore filters. The Waters semi-preparative HPLC instrument (Waters 2535 Quaternary Gradient Module) equipped with a diode array detector (Waters 2996) was used. Compounds were separated using a Supelco Discovery BIO WidePore C18 column (250 x 10 mm, 5 µm particle size).

System I: Linear gradients of eluent A (0.1% (v/v) TFA in bidistilled water) and eluent B (0.1% (v/v) TFA in acetronitrile) were applied according to the following method: 0-20 min (5-100% B), 20-25 min (100% B), 25-26 min (100-5% B), 26-30 min (5% B). Elution was monitored via absorbance using diode array (200-700 nm) with a flow of 3 ml/min.

System II: Linear gradients of eluent A (0.1% (v/v) TFA in bidistilled water) and eluent B (0.1% (v/v) TFA in acetronitrile) were applied according to the following method: 0-25 min (20-70% B), 25-27 min (70% B), and 27-29 min (70-20% B) and 29-30 min (20% B). Elution was monitored using diode array (200-700 nm) with a flow of 3 ml/min.

5 Radio-HPLC

The Radio-HPLC analyses were performed using HPLC grade solvents and bi-distilled H_2O filtered through 0.22 µm Millipore filters. Different systems and methods were used in the HPLC analyses as described below.

Radiometric System I: Perkin-Elmer LC 200 analytical HPLC coupled to a LC 290 UV/Vis detector and to a Berthold LB-507 A radiometric detector. Compounds were separated using a Macherey-Nagel EC250/4 Nucleosil 100-10 C18 column (250 x 4 mm, 10 Å pore size, 10 µm particle size). Linear gradients of eluent A (0.1% (v/v) TFA in bidistilled water) and eluent B (0.1% (v/v) TFA in acetronitrile) were applied according to the following method: 0-5 min (5% B), 5-25 min (5-100% B), 25-26 min (5% B), and 26-30 min (5% B). Elution was monitored via absorbance at 220 nm or γ-detection with a flow of 1 ml/min.

Radiometric System II: Shimadzu LC-10 AD pumps system coupled to UV/Vis detector SPD-10A and to a RayTest GABI radiometric detector. Compounds were separated using a Kinetex C18 - 100 Å pore size column (150 mm x 4.6 mm, 5 µm particle size). Linear gradients of eluent A (0.1% (v/v) TFA in bidistilled water) and eluent B (0.1% (v/v) TFA in acetronitrile) were applied according to the following method: 0 min (5% B), 0-20 min (5-100% B), 20-25 min (100% B), and 25-30 min (5% B). Elution was monitored via absorbance at 220 nm, 280 nm and γ-detection with a flow of 1 ml/min.

6 Instant Thin Layer Chromatography (ITLC)

Radiolabelling yields and radiochemical purity were evaluated by ITLC using silica-gel layers (ITLC-SG, Agilent) eluted using a mixture of H_2O:MeOH (7:3 v/v) as mobile phase. The radioactivity distribution in the chromatograms was detected in a Raytest γ-miniGITA TLC scanner.

7 Chemical Synthesis

7.1 Synthesis of the derivative Tz (5)

The derivative Tz (**5**) (as shown in Scheme 1) was obtained by first inserting a Boc protecting group on the benzylic amine of the commercially available 4-(aminomethyl)benzonitrile hydrochloride to afford the corresponding *tert*-butyl 4-cyanobenzylcarbamate (**7**). The reaction, performed in aqueous conditions, allowed to obtain the desired product in nearly quantitative yield. The tetrazine ring was then synthesized by slight modification of a Lewis acid catalyzed procedure described in the literature. The anhydrous hydrazine unavailable in Europe was replaced with monohydrated hydrazine.(2,3) The reaction was performed inside sealed tubes containing a stirring mixture of formamidine acetate, the *tert*-butyl 4-cyanobenzylcarbamate (**7**) and a catalytic amount of $Zn(OTf)_2$ in dry 1,4-dioxane. The monohydrated hydrazine was then added to the 1,4-dioxane suspension with the use of a syringe, and the mixture was left stirring at 60 °C overnight. The following day, the di-hydrotetrazine (**8**) was oxidized with $NaNO_2$ in acidic conditions. After the purification of the compound by silica gel column chromatography, the *tert*-butyl 4-(1,2,4,5-tetrazin-3-yl)benzylcarbamate (**9**) was successfully isolated.

The deprotection of the Boc group was achieved with a standard procedure using trifluoroacetic acid and DCM to afford the desired Tz (**5**) derivative.

7.1.1 Synthesis of *tert*-Butyl 4-cyanobenzylcarbamate (7)

The commercially available 4-(aminomethyl)benzonitrile hydrochloride (1 g, 6 mmol) was dissolved into 5 mL of H_2O and then added to a stirring solution of NaOH 0.75 g and Boc_2O (1.5 g, 1.1 eq.) in 5 mL of H_2O. After stirring the mixture during 16 h at room temperature, the white precipitate formed was filtered, washed with 50 mL of H_2O and dried under reduced pressure to afford 1.34 g of the desired product as a white powder in 94% yield.(4) **ESI-MS:** $C_{13}H_{17}N_2O$ $[M+H]^+$ 233.1, found 233. 4 and $[M+Na]^+$ 255.1 found 255.4. **^{1}H-NMR:** (300 MHz, $CDCl_3$) 1.45 (s, 9H), 4.35 (d, 2H, J=4.1 Hz), 5.00 (bs, 1H), 7.36 (d, 2H, J=7.9 Hz), 7.60 (d, 2H, J=7.1 Hz).

7.1.2 Synthesis of *tert*-butyl 4-(1,2,4,5-tetrazin-3-yl)benzylcarbamate (9)

Inside a glass tube with a magnetic stirrer were added *tert*-butyl 4-cyanobenzylcarbamate (98 mg, 0.44 mmol), formamidine acetate (439 mg, 9.6 eq.), and the $Zn(OTf)_2$ metal catalyst (77 mg, 0.5 eq.). The tube was sealed with the cap and the air removed by purging with N_2. Then, 0.5 mL of dry 1,4-dioxane was added and the suspension was stirred before adding, with the help of a syringe, the hydrazine monohydrate (1 mL, 218 eq.). The mixture was heated at 60°C and left stirring overnight. The day after, the tube was cooled down and diluted by addition of 10 mL of EtOAc. The reaction mixture was washed with 10 mL of 1M HCl and the aqueous phase back extracted with EtOAc (3 x 5 mL). The reunited organic phases were dried over $MgSO_4$ and concentrated in the rotavapor. The crude residue (**8**, not isolated) was then dissolved in a 1:1 (v/v) mixture of DCM and acetic acid (5 mL) and $NaNO_2$ was slowly added over 15 minutes (584 mg). The reaction turned red and developed toxic nitrous fumes and, therefore, was carefully performed in a well-ventilated fume hood. Finally, the reaction mixture was diluted with DCM (25 mL) and washed with 25 mL of a saturated solution of $NaHCO_3$ in water. The aqueous phase was back extracted with DCM (3 x 10 mL) and the reunited organic phases were dried over $MgSO_4$ and concentrated in the rotavapor. The pink crude was successively purified by silica gel chromatography using as mobile phase a mixture of DCM containing 2% of Et_2O, to afford the desired product in yields ranging from 10 to 40%(4). **ESI-MS:** $C_{14}H_{17}N_5O_2$ $[M+H]^+$ calculated 288.1, found 289.6. **^{1}H-NMR:** (300 MHz, $CDCl_3$) 1.48 (s, 9H), 4.45 (bs, 2H), 4.98 (bs, 1H), 7.51 (d, 2H, J=7.7 Hz), 8.60 (d, 2H, J=7.7 Hz), 10.2 (s, 1H).

7.1.3 Synthesis of [4-(1,2,4,5-tetrazin-3-yl)phenyl]methanamine (5)

Inside a flask, the *tert*-butyl 4-(1,2,4,5-tetrazin-3-yl)benzylcarbamate (**9**) (37 mg, 0.13 mmol) was dissolved in 2 mL of DCM and then 2 mL of TFA were added. The mixture was stirred 2 hours at room temperature and the progress of the reaction was monitored by TLC. When the starting material was no longer visible, the solvent and TFA were evaporated under reduced pressure to afford the desired product [4-(1,2,4,5-tetrazin-3-yl)phenyl]-methanamine as a pink TFA salt in quantitative yield. **ESI-MS:** $C_9H_9N_5$ $[M+H]^+$ calculated 188.1, found: 188.2. **^{1}H-NMR:** (400 MHz, MeOD) 4.22 (bs, 2H), 4.27 (bs, 2H), 7.73 (d, 2H, J=7.4 Hz), 8.66 (d, 2H, J=7.4 Hz), 10.38 (s, 1H). **^{13}C-NMR:** (400 MHz, MeOD) 42.5, 128.4, 129.5, 132.9, 137.8, 158.1, 165.9.

7.2 Synthesis of the pegylated derivative Tz-PEG (6)

The pegylated tetrazine derivative Tz-PEG (**6**) (as shown in Scheme 2), was obtained by initially reacting the commercially available 4-(aminomethyl)benzonitrile hydrochloride with glutaric anhydride to afford the 5-(4-(cyano)benzylamino)-5-oxopentanoic acid (**10**). The tetrazine ring was

then obtained using a similar metal-catalyzed procedure, in the presence of $Ni(OTf)_2$.(5) The oxidation of the di-hydrotetrazine was performed in acidic conditions by addition of $NaNO_2$ to afford the 5-(4-(1,2,4,5-tetrazin-3-yl)benzylamino)-5-oxopentanoic acid intermediate (**11**). This intermediate was then coupled with the pegylated linker, using PyBOP to activate the carboxylic acid in the presence of DIPEA, yielding the *tert*-butyl(1-(4-(1,2,4,5-tetrazin-3-yl)phenyl)-3,7-dioxo-11,14,17,20,23,26,29,32,35,38-41-undecaoxa-2,8-diazatri-tetracontan-43-yl)carbamate (**12**). The pegylated linker, with an intermediate length, was expected to modulate the pharmacokinetics of the resulting radiocomplexes, namely by increasing their blood half-lives. The final Boc deprotection of the amino group was achieved with a 1:1 mixture (v/v) of TFA in DCM and the final product Tz-PEG (**6**) was purified by HPLC using the Semi-Preparative System II.

7.2.1 Synthesis of 5-(4-(cyano)benzylamino)-5-oxopentanoic acid (10)

The commercially available 4-(aminomethyl)benzonitrile hydrochloride (113 mg, 0.67 mmol) was purged with N_2 in a dry flask before adding 5 mL of ACN and NEt_3 (0.103 mL, 1.1 eq.). Then, the glutaric anhydride was added (77 mg, 1 eq.) and the reaction was heated to reflux (85 °C) overnight. The day after the flask was cooled down, 1 mL of distilled water was added to the crude and the mixture acidified to pH 3 using 1M HCl. The aqueous phase was extracted with EtOAc (3x5 mL) and the reunited organic phases dried over $MgSO_4$ and then concentrated in the rotavapor to afford the desired product as a white powder in quantitative yield.(5) **ESI-MS:** $C_{13}H_{14}N_2O_3$ $[M+H]^+$ calc. 247.1, found: 247.8 and $[M+Na]^+$ calc. 269.1, found: 269.4. **^{1}H-NMR:** (400 MHz, CD_3CN) 1.82 (pentet, 2H), 2.25 (t, 2H, J=7.4 Hz), 2.30 (t, 2H, J=7.2 Hz), 4.40 (d, 2H, J=6.1 Hz), 7.02 (bs, 1H), 7.42 (d, 2H, J=7.9Hz), 7.68 (d, 2H J=7.7 Hz).

7.2.2 Synthesis of 5-(4-(1,2,4,5-tetrazin-3-yl)benzylamino)-5-oxopentanoic acid (11)

Inside a flask, were introduced the 5-(4-(cyano)benzylamino)-5-oxopentanoic acid (**10**) (167 mg, 0.67 mmol), the formamidine acetate (283 mg, 4 eq.), the $Ni(OTf)_2$ and the flask purged with N_2. Then, the hydrazine monohydrate was added (0.6 mL, 18 eq.) and the mixture was left stirring overnight at room temperature. The day after, the pH of the mixture was brought to 3 using 1M HCl and then $NaNO_2$ was added (468 mg, 10 eq.). The mixture turned pink and was extracted using DCM (3x10 mL) and the reunited organic phases were dried over $MgSO_4$ and then concentrated in the rotavapor. The pink crude was then purified by silica gel chromatography (R_f = 0.3) using as a mobile phase a mixture of DCM and MeOH (95:5) (v/v) with 3 drops of TFA allowing to recover the desired product as a pink solid in 22% yield.(5) **ESI-MS:** $C_{13}H_{14}N_5O_3$ $[M+H]^+$ calc. 302.1, found 302.7 and $[M+Na]^+$ calc. 324.1, found 324.5. **^{1}H-NMR:** (400 MHz, MeOD) 1.94 (pentet, 2H), 2.35 (t, 4H, J=7.4 Hz), 4.52 (s, 2H), 7.57 (d, 2H, J=8.2 Hz), 8.56 (d, 2H, J=8.3 Hz), 10.33 (s, 1H). **^{13}C-NMR:** (400 MHz, MeOD) 20.9, 32.8, 34.7, 42.4, 127.9, 130.9, 144.2, 157.8, 166.4, 174.1, 175.5.

7.2.3 Synthesis of Tz-PEG-Boc (12) (*tert*-butyl(1-(4-(1,2,4,5-tetrazin-3-yl)phenyl)-3,7-dioxo-11,14,17,20,23,26,29,32,35,38,41-undecaoxa-2,8-diazatritetra-contan-43-yl)carbamate)

In a flask, the 5-(4-(1,2,4,5-tetrazin-3-yl)benzylamino)-5-oxopentanoic acid (**11**) (32 mg, 0.1 mmol) was dissolved in dry DMF and then PyBOP (58.1 mg, 1.05 eq.) and DIPEA (174.5 µL, 10 eq.) were added. After 5 minutes, NH_2-PEG_{11}-NHBoc (75.4 mg, 1.1 eq.) was added to the mixture and the reaction was left stirring overnight at room temperature with the flask protected from the light using aluminium paper. The day after the solvent was evaporated under reduced pressure and the crude mixture purified using a C-18 Sep-Pak cartridge. The reactants were washed away using 4 - 8% of ACN in H_2O (with 0.1% of TFA) and the desired product was successively eluted with pure ACN and obtained in 80% yield as a pink oil. **ESI-MS:** $C_{43}H_{73}N_7O_{15}$ $[M+Na]^+$ calculated 950.5, found 951.2.

^{1}H-NMR: (400 MHz, MeOD) 1.44 (s, 9H), 1.82 (pentet, 2H), 2.29 (t, 2H, J=7.6 Hz), 2.35 (t, 2H, J=7.4 Hz), 3.19 (m, 2H), 3.24 (m, 2H), 3.34 (m, 22H) 3.64 (m, 22H) 4.54 (s, 2H), 7.59 (d, 2H, J=8.3 Hz), 8.58 (d, 2H, J=8.4 Hz), 10.36 (s, 1H).

7.2.4 Synthesis of Tz-PEG (6) (N_1-(4-(1,2,4,5-tetrazin-3-yl)benzyl)-N_5-(35-amino-3,6,9,12,15,18,21,24,27,30,33-undecaoxapentatriacontyl)-glutaramide)

Inside a flask, the Tz-PEG-Boc (**12**) was dissolved in 10 mL of DCM and then 2 mL of TFA were added. The mixture was stirred 1 hour at room temperature and the progress of the reaction was monitored by HPLC. When the starting material was no longer visible, the solvent and TFA were evaporated under reduced pressure. The crude mixture was then separated using the HPLC Semi Preparative System II. The final product (R_t = 13.8 minutes) was recovered, after lyophilization, in good chemical purity as a pink oil in 50% yield. **ESI-MS:** $C_{38}H_{65}N_7O_{13}$ $[M+H]^+$ calculated 828.5, found 828.9. **^{1}H-NMR:** (400 MHz, MeOD) 1.86 (pentet, 2H), 2.27 (t, 2H, J=7.6 Hz), 2.33 (t, 2H, J=7.4 Hz), 3.17 (m, 2H), 3.34 (m, 22H) 3.65 (m, 22H) 4.52 (s, 2H), 7.58 (d, 2H, J=8.0 Hz), 8.57 (d, 2H, J=8.0 Hz), 10.35 (s, 1H).

7.3 Synthesis of the Clickable Chelators

7.3.1 Synthesis of DOTA-Tz (1)

In a flask under N_2 inert atmosphere, the Tz derivative (**5**) (17 mg, 0.09 mmol) was introduced together with the DOTA-NHS (55 mg, 0.8 eq.). Then, 1 mL of dry DMF and NEt_3 (125 µL, 10 eq.) were added and the reaction mixture was stirred at room temperature (rt) during 2 hours. After the completion of the reaction, the mixture was concentrated under reduced pressure and the crude product purified using the HPLC Semi-Preparative System I. The final product (R_t = 13.4 minutes) was recovered, after lyophilization, as a pink solid in 7% yield. **ESI-MS:** $C_{25}H_{35}N_9O_7$ $[M+H]^+$ calculated 574.3, found 574.7. **^{1}H-NMR:** (400 MHz, D_2O) 3.18 (m, 8H), 3.40 (m, 8H), 3.66-3.83 (m, 8H), 4.52 (s, 2H), 7.61 (d, 2H, J=8.2 Hz), 8.44 (d, 2H, J=8.2 Hz), 10.38 (s, 1H).

7.3.2 Synthesis of DOTAGA-Tz (2)

In a flask under N_2 inert atmosphere, the Tz derivative (**5**) (23 mg, 0.12 mmol) was introduced together with the DOTAGA anhydride (55 mg, 1 eq.). Then, 1 mL of dry DMF and NEt_3 (167 µL, 10 eq.) were added and the reaction mixture was stirred at rt for 3 hours. After the completion of the reaction, the mixture was concentrated under reduced pressure and the crude product purified using a C-18 Sep-Pak cartridge by elution with H_2O containing 0.1% of TFA and increasing percentages of MeOH (increments of 25%). The collected fractions were analyzed by HPLC (Radiometric System I) and the fraction containing the desired conjugate was identified by ESI-MS. After lyophilization, the desired compound (R_t = 15.1 minutes) was recovered as a pink powder in 4% yield. **ESI-MS:** $C_{28}H_{39}N_9O_9$ $[M-H]^-$ calculated 644.3, found 644.2. **^{1}H-NMR:** (400 MHz, MeOD) 2.05 (m, 2H), 2.15 (m, 2H), 2.67-2.77 (m, 4H), 3.05 (m, 4H), 3.23 (m, 4H), 3.37 (s, 6H), 3.54 (m, 4H), 4.11 (m, 1H), 4.86 (s, 2H), 7.58 (d, 2H, J=8.0 Hz), 8.56 (d, 2H, J=7.70 Hz), 10.34 (s, 1H).

7.3.3 Synthesis of DOTA-PEG-Tz (3)

In a flask under N_2 inert atmosphere, the Tz-PEG derivative (**6**) (18 mg, 0.02 mmol) was introduced together with the DOTA-NHS (16 mg, 1 eq.). Then, 3 mL of dry DMF and NEt_3 (28 µL, 10 eq.) were added and the reaction mixture was stirred at rt for 3 hours. After the completion of the reaction, the mixture was concentrated under reduced pressure and the crude product purified using the HPLC Semi-

Preparative System I. After lyophilization, the final product (R_t = 14.3 minutes) was recovered in good chemical purity as a pink oil in 20% yield. **ESI-MS:** $C_{54}H_{91}N_{11}O_{20}$ [M-H]$^-$ calculated 1212.6, found 1212.6. **^{1}H-NMR:** (400 MHz, MeOD) 1.96 (m, 2H), 2.26 (m, 4H), 3.37 (m, 30H), 3.55 (m, 35H), 3.87 (m, 6H), 4.51 (s, 2H), 4.86 (s, 2H), 7.57 (d, 2H, J=8.1 Hz), 8.55 (d, 2H, J=8.4 Hz), 10.34 (s, 1H).

7.3.4 Synthesis of DOTAGA-PEG-Tz (4)

In a flask under N_2 inert atmosphere, the Tz-PEG derivative (**6**) (23 mg, 0.03 mmol) was introduced together with the DOTAGA anhydride (13 mg, 1 eq.). Then, 2 mL of dry DMF and NEt_3 (39 µL, 10 eq.) were added and the reaction mixture was stirred at rt overnight. After the completion of the reaction, the mixture was concentrated under reduced pressure and the crude product purified using the HPLC Semi-Preparative System I. After lyophilization, the final product (R_t = 14.8 minutes) was recovered as a pink oil in 20% yield. **ESI-MS:** $C_{57}H_{95}N_{11}O_{22}$ [M+H]$^+$ calculated 1286.7, found 1287.2.

7.4 Synthesis of the Clickable Bombesin Antagonists

7.4.1 Synthesis of AR-PEG$_4$-TCO

The peptide AR with the sequence D-Phe-Gln-Trp-Ala-Val-Gly-His-Sta-Leu-NH_2 was obtained by solid phase peptide synthesis (SPPS) methodologies employing a standard Fmoc strategy performed in a CEM 12-Channel Automated Peptide Synthesizer, using microwave irradiation and rink amide resin (MBHA). Cleavage of the peptide from the Rink resin was achieved in acidic conditions using a mixture of TFA/Me_3Si/H_2O (2 mL/50 µL/50 µL) for 8 mg of Rink resin during 1 hour at room temperature. The solution was concentrated under nitrogen to 5 % of the initial volume and the desired product was precipitated by addition of ice-cooled Et_2O (10 volumes). The precipitate was recovered upon centrifugation, identified by ESI-MS analysis and purified by HPLC. Then, 3.1 mg (2.7 µmol) of the AR peptide were dissolved in 3 mL of dry DMF and 2 µL of DIPEA were added. The mixture was stirred for 5 minutes at room temperature before the addition of TCO-PEG4-NHS (2.9 mg, 2.1 eq.). The solution was stirred for 48 hours at room temperature and under N_2 atmosphere. The solvent was evaporated under vacuum and the resulting peptide AR-PEG4-TCO was purified by HPLC (Radiometric System I, R_t = 16.4 min) and successively lyophilized to afford the desired compound in 57 % yield. **ESI-MS**: $C_{75}H_{113}N_{15}O_{18}$ [M+H]$^+$ calculated 1512.8, found 1512.8.

7.4.2 Synthesis of DOTA-Tz-TCO-PEG4-AR and Radiolabelling with ^{90}Y and ^{111}In

The derivatives AR-PEG$_4$-TCO (303 µg, 200 nmol) and DOTA-Tz (100 µg, 1.5 eq.) were dissolved in water to a final volume of 320 µL and incubated at 37 °C during 1 hour and half. After completion of the reaction, the crude mixture was purified by HPLC (Radiometric System I, R_t = 13.9 min) to isolate the desired DOTA-Tz-TCO-PEG$_4$-AR conjugate in 38% yield. **ESI-MS:** $C_{100}H_{146}N_{22}O_{25}$ [M+2H]$^{2+}$ calculated 1029.6, found 1029.6.

The radiolabelling of the conjugate DOTA-Tz-TCO-PEG$_4$-AR with ^{90}Y was performed in the presence of ascorbic acid, a convenient radical scavenger that is ideal for the radiolabelling with β^- emitters such as ^{90}Y because of its protective role against the autoradiolysis issues of radiolabelled compounds. The radiolabelling reaction was performed at 95 °C during 15 minutes to afford the radioconjugate [^{90}Y]Y-DOTA-Tz-TCO-PEG$_4$-AR in radiochemical yields above 98% and excellent specific activities up to 52 MBq/nmol. The compound was characterized by HPLC analysis using the Radiometric System I (R_t = 13.91, γ-detection) as shown in Figure SM 6.

The radioconjugate did not require any further purification and was stable at room temperature, with no radiolysis issues detected up to 24 hours after the radiolabelling as shown in Figure SM 7A. The incubation of the compound [^{90}Y]Y-DOTA-Tz-TCO-PEG$_4$-AR at 37 °C in human serum (HS) during six days revealed excellent in vitro stability as determined by ITLC radiochromatogram eluted with citrate buffer (pH 5.5, 0.1 M), as shown in Figure SM 7B.

The clickable radiocomplex ^{111}In-DOTAGA-Tz was reacted with the functionalized AR-PEG4-TCO peptide and the resulting radioconjugate [^{111}In]In-DOTA-Tz-TCO-PEG$_4$-AR was analyzed by HPLC. The reaction proceeded to the completion with the complete disappearance of the peak corresponding to the radiocomplex ^{111}In-DOTAGA-Tz in the chromatogram obtained by γ-detection. The final radiopeptide was characterized by HPLC analysis using the Radiometric System I (R_t = 19.2 min, γ-detection) as shown in Figure SM 8.

8 ^{nat}In-complexes

The four clickable compounds DOTA-Tz, DOTAGA-Tz, DOTA-PEG-Tz and DOTAGA-PEG-Tz were reacted with 4 equivalents of anhydrous $InCl_3$ in 0.4 M acetate buffer at pH 5.5 and heating at 80°C for 15 minutes. The purification to remove the excess of $InCl_3$ was achieved using C-18 Sep-Pak cartridges eluted with H_2O containing 0.1% of TFA and increasing percentages of ACN with 0.1% TFA (increments of 25%). Because of the difficult and low-yielding chemical synthesis of the clickable ligands, the cold complexes were prepared in very small quantities (1-2 mg) just to perform their analytical characterization by HPLC (Radiometric System I) and ESI-MS and confirm the chemical identity of the clickable [^{111}In]In-radiocomplexes.

In-DOTA-Tz (R_t = 12.5 min, ESI-MS: $InC_{25}H_{32}N_9O_7$ $[M+H]^+$ calc. 686.1, found 686.7).

In-DOTAGA-Tz (R_t = 14.8 min, ESI-MS: $InC_{28}H_{36}N_9O_9$ $[M+H]^+$ calc. 758.2, found 758.7).

In-DOTA-PEG-Tz (R_t = 16.2 min, ESI-MS: $InC_{54}H_{88}N_{11}O_{20}$ $[M+H]^+$ calc. 1326.5, found 1327.2).

In-DOTAGA-PEG-Tz (R_t = 16.4 min, ESI-MS: $InC_{57}H_{92}N_{11}O_{22}$ $[M-H]^-$ calc. 1396.5, found 1396.5).

9 In vivo Studies

9.1 Biodistribution in Normal Mice

The in vivo behavior of the [^{111}In]In-radiolabelled complexes was evaluated in groups of 3 female CD-1 mice (randomly bred, Charles River) weighting approximately 25 g each. Animals were injected intravenously with 100 μL (0.8 – 3.3 MBq/ 30 – 120 μCi) of each preparation via the tail vein. The animals were housed in a temperature- and humidity-controlled room with a 12 h light/12 h dark schedule and maintained on normal diet ad libitum. Mice were sacrificed by cervical dislocation at different time points (15 and 60 min) post-injection (p.i.). The injected radioactive dose and the radioactivity remaining in the animal after sacrifice were measured in a dose calibrator. The difference between the radioactivity in the injected and sacrificed animal was assumed to be due to total excretion

from whole animal body. Blood samples were taken by cardiac puncture at sacrifice. Tissue samples of the main organs were then dissected, weighted and counted in a gamma counter (Berthold). Biodistribution results were expressed as percentage of the injected dose per gram tissue (%I.D./g) in the form of an average with standard deviation (%ID/g; mean ± standard deviation (SD).

9.2 Biodistribution in PC3 bearing mice

Male, 5-6 weeks old NodCB17 SCID mice were obtained from Janvier Lab., France. Mice were housed under pathogen-free conditions with food and water ad libitum, and a 12-12 hour light-dark cycle. Veterinarian staff and investigators observed the mice daily to ensure animal welfare and determine if human endpoints (e.g., hunched and ruffled appearance, apathy, ulceration, severe weight loss, tumour burden) were reached. The mice were subcutaneously injected in the shoulder with 200 µL bolus containing a suspension of 10^6 freshly harvested human prostatic cell line PC3 in Matrigel™ Basement Membrane Matrix (BD Sciences) under anaesthesia with 2% isoflurane. The animals were kept under pathogen-free condition and experiments were performed 2–3 weeks later when a tumour reached a volume of approximate 390 ± 190 mm^3. Animals were allocated into treatment groups using a complete randomisation.

The examination of physiological distribution for [^{90}Y]Y-DOTA-Tz were carried out on 4 mice (n = 4). For the control bolus, 0.061 nmol, 0.1 mL, 7.5 µCi of [^{90}Y]Y-DOTA-Tz was intravenously injected by tail vein. The examination of physiological distribution for [^{90}Y]Y-DOTA-Tz-TCO-PEG_4-AR were carried out on groups of 8 mice (n = 4). For the control bolus, 0.061 nmol, 0.1 mL, 7.5 µCi of ^{90}Y-DOTA-Tz TCO-PEG_4-AR was intravenously injected by tail vein. At appropriate time points (15 min, 1h or 2h after-injection), the animals were euthanized, tissues of interest were dissected, weighted, and their radioactivity was measured using NaI gamma counter supplied with adapter for the whole-body measurement.

Biodistribution of ^{111}In-DOTA-Tz-TCO-PEG_4-AR was performed by intravenous injection of 100 µL (50 – 75 µCi) of the preparation into mice (n =4) via the tail vein. Immediately after injection, radioactive dosage administered into each animal was measured in a dose calibrator (Capintec CRC25R). For the in vivo click chemistry studies in the same PC3 xenografts (n=3), a bolus of 1 nmol of TCO-PEG4-AR, dissolved in 0.1 mL of saline, was injected in the tail vein, and after 4h the [^{111}In]In-DOTA-Tz (0.1mL, 50 – 75 µCi µCi). After 1 h of administration of the radioactive preparations, the mice groups were sacrificed by cervical dislocation and the radioactivity in the sacrificed animal was measured. The difference between the radioactivity in the injected and the sacrificed animal was assumed to be due to total excretion. The animals were weighed and dissected. Then, tissues of interest were removed, weighed and counted in a γ-counter (Berthold, LB211, Germany).

For all the performed biodistribution studies, selected organs and tissues were assayed for their radioactivity and weighted. The physiological distribution was then calculated and expressed in terms of the percentage of the administrated radioactivity found in each of the selected organs or tissues per gram (%ID/g) in the form of an average with standard deviation (%ID/g; mean ± standard deviation (SD)).

10 Stability Studies

10.1 [^{111}In]In-Radiocomplexes: In vitro and in vivo stability

The in vitro stability of the radiocomplexes was studied by ITLC and HPLC analysis of aliquots of the radiolabelled compound dissolved in different media. For the evaluation of the stability in PBS, Cell Culture Media and Human Serum a suitable volume of the radioactive complexes was diluted into 4 times the volume of the desired medium. The mixtures were incubated at 37 °C and aliquots were taken at the different time points and analysed by HPLC and ITLC. The radiocomplexes incubated in Human Serum were first treated with a four-times bigger volume of cold ethanol to induce protein precipitation. After centrifugation during 10 minutes at 5000 rpm, the supernatants were separated from the pellets and analysed by HPLC.

Blood samples from mice injected with the tested radiocomplexes, collected at sacrifice time, were centrifuged, the serum separated and treated with ethanol (1:2 proportion) to precipitate the proteins and the supernatant was analyzed by HPLC for stability evaluation. The urine samples were centrifuged at 3000 rpm for 10 min and the supernatant was also analyzed by HPLC.

10.2 [^{90}Y]Y-DOTA-Tz-TCO-PEG$_4$-AR and [^{111}In]In-DOTA-Tz-TCO-PEG$_4$-AR: In vitro Stability

The preparations of the radioconjugate [^{90}Y]Y-DOTA-Tz-TCO-PEG$_4$-AR was stable up to 24 hours after the radiolabelling at room temperature as verified by HPLC analysis (Figure SM 7A). The HPLC chromatogram did not shown any radiolysis issue and the in vitro stability of the radioconjugates ^{90}Y-DOTA-Tz-TCO-PEG$_4$-AR was further evaluated also by incubation at 37 °C in human serum (HS) during six days by ITLC analysis. As shown in Figure SM 7B, the radioconjugate exhibited an excellent radiochemical stability, with no issues of autoradiolysis or degradation upon interaction with the plasma proteins detected over six days of incubation by ITLC. The radiochemical purity measured by autoradiography of ITLC strips eluted using citrate buffer (pH 5.5, 0.1 M) was found above 99 % even after 6 days of incubation in the presence of HS.

The *in vitro* stability of the radioconjugate [^{111}In]-DOTA-Tz-TCO-PEG$_4$-AR was evaluated by incubation at 37 °C in cell culture media (CCM), in human serum (HS) and in PBS during 24 hours. The radioconjugate exhibited excellent stabilities in CCM, with no degradation issues up to 24 hours of incubation.

11 Lipophilicity Determination

The octanol-water partition coefficient ($P_{o/w}$) of the radiocomplexes were determined by the "shake-flask" method.(6) The radiolabelled conjugate (20 µL) was added to a mixture of PBS pH 7.4 (1 mL) and 1-octanol (1 mL) previously saturated in each other by vigorous stirring. The mixture was vortexed and centrifuged (3000 rpm, 10 min, RT) to allow phase separation. After phase separation, aliquots (100 µL) of both organic and water phases were counted in a gamma counter and the ratio between the radioactivity in both phases ($P_{o/w}$) was calculated and results expressed as log $P_{o/w}$.

12 Schemes

Scheme 1. Synthesis of the clickable derivative Tz (**5**)

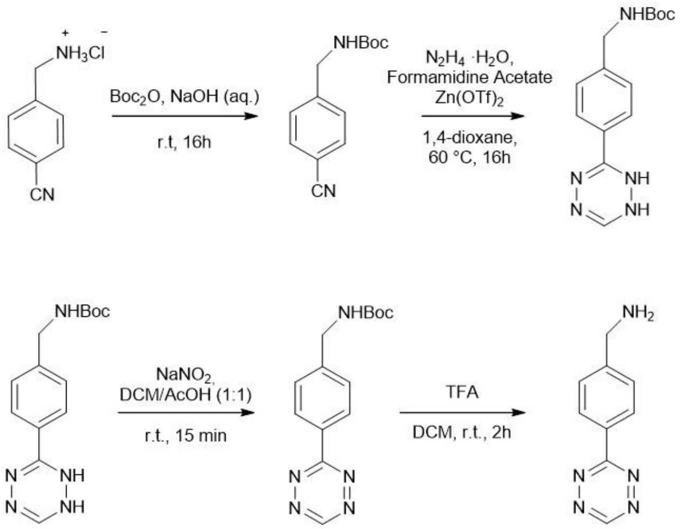

Scheme 2. Synthesis of the clickable pegylated derivative Tz-PEG (**6**)

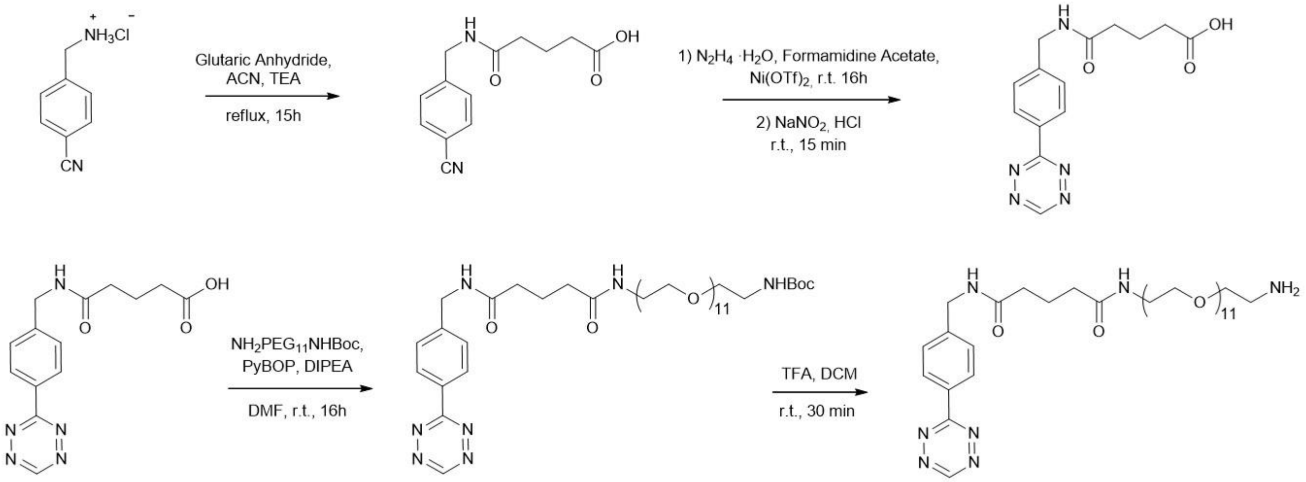

13 Tables

Table 1. Synthetic yields, R_t and ESI-MS m/z values for the four clickable ligands and the respective cold ^{nat}In complexes.

Derivative	Yield%	R_t - HPLC (min)	Chemical Formula (Exact Mass)	ESI-MS (m/z)
**DOTA-Tz**	7	13.4	$C_{25}H_{35}N_9O_7$ (574.3)	574.7 (positive mode)
**DOTAGA-Tz**	4	15.1	$C_{28}H_{39}N_9O_9$ (645.3)	644.2 (negative mode)
**DOTA-PEG-Tz**	20	14.3	$C_{54}H_{91}N_{11}O_{20}$ (1213.6)	1212.6 (negative mode)
**DOTAGA-PEG-Tz**	22	14.8	$C_{57}H_{95}N_{11}O_{22}$ (1285.7)	1287.2 (positive mode)
**In-DOTA-Tz**	-	12.5	$InC_{25}H_{32}N_9O_7$ (685.1)	686.7 (positive mode)
**In-DOTAGA-Tz**	-	14.9	$InC_{28}H_{36}N_9O_9$ (757.2)	758.7 (positive mode)
**In-DOTA-PEG-Tz**	-	16.2	$InC_{54}H_{88}N_{11}O_{20}$ (1325.5)	1327.2 (negative mode)
**In-DOTAGA-PEG-Tz**	-	16.4	$InC_{57}H_{92}N_{11}O_{22}$ (1397.5)	1396.5 (negative mode)

Table 2. Radiochemical yields and HPLC retention times of the clickable [^{111}In]In-radiocomplexes.

Derivative	RCY%	HPLC Purification	R_t - HPLC (min) γ-detection
**[^{111}In]In-DOTA-Tz**	80	Yes	12.2
**[^{111}In]In-DOTAGA-Tz**	80	Yes	15.1
**[^{111}In]In-DOTA-PEG-Tz**	95	No	16.6
**[^{111}In]In-DOTAGA-PEG-Tz**	95	No	16.2

Table 3. Biodistribution data (%I.D./g ± SD) of the [^{111}In]In-radiocomplexes 15 min and 1 h p.i. (n = 3 for all time points except * where, n = 5)

Organs	[^{111}In]In-DOTA-Tz		[^{111}In]In-DOTA-PEG-Tz		[^{111}In]In-DOTAGA-Tz		[^{111}In]In-DOTAGA-PEG-Tz	
	**15 min**	**1 h**	**15 min**	**1 h**	**15 min**	**1 h***	**15 min**	**1 h***
**Blood**	2.5 ± 1.4	0.32 ± 0.07	3.1 ± 0.4	0.44 ± 0.07	3.2 ± 0.7	1.1 ± 0.3	3.7 ± 0.6	0.7 ± 0.4
**Liver**	2.3 ± 0.8	0.29 ± 0.09	1.1 ± 0.2	0.70 ± 0.30	1.6 ± 0.4	1.0 ± 0.3	0.8 ± 0.1	0.3 ± 0.2
**Intestine**	0.3 ± 0.2	0.05 ± 0.01	0.32 ± 0.05	0.05 ± 0.01	0.4 ± 0.1	0.26 ± 0.09	0.31 ± 0.05	0.4 ± 0.2
**Spleen**	0.8 ± 0.2	0.09 ± 0.01	0.66 ± 0.28	0.18 ± 0.04	0.6 ± 0.1	0.3 ± 0.1	0.6 ± 0.2	0.11 ± 0.05
**Heart**	0.8 ± 0.5	0.11 ± 0.02	1.0 ± 0.2	0.15 ± 0.08	0.9 ± 0.2	0.4 ± 0.1	1.2 ± 0.6	0.13 ± 0.08
**Lung**	1.8 ± 0.9	0.29 ± 0.03	1.6 ± 0.3	0.36 ± 0.04	1.9 ± 0.4	0.8 ± 0.2	1.4 ± 0.2	0.3 ± 0.2
**Kidney**	5.2 ± 3.3	1.6 ± 0.3	5.8 ± 1.3	1.9 ± 0.3	7.2 ± 0.4	3.6 ± 1.0	4.8 ± 0.7	2.2 ± 0.6
**Muscle**	1.1 ± 0.9	0.08 ± 0.01	0.9 ± 0.2	0.10 ± 0.02	0.9 ± 0.2	0.27 ± 0.07	0.86 ± 0.07	0.2 ± 0.1
**Bone**	1.0 ± 0.7	0.12 ± 0.03	0.78 ± 0.08	0.11 ± 0.01	1.2 ± 0.2	0.5 ± 0.1	0.8 ± 0.2	0.17 ± 0.09
**Stomach**	0.3 ± 0.2	0.09 ± 0.05	0.5 ± 0.3	0.09 ± 0.02	0.6 ± 0.3	0.3 ± 0.2	1.0 ± 0.5	1.1 ± 0.8
**Pancreas**	0.4 ± 0.2	0.06 ± 0.01	0.53 ± 0.07	0.09 ± 0.01	0.6 ± 0.1	0.3 ± 0.1	0.53 ± 0.05	0.12 ± 0.07
**Excretion (% I.D.)**	58.3 ± 25.3	91.8 ± 1.0	65.3 ± 5.4	93.6 ± 0.8	62.7 ± 1.5	81.5 ± 5.1	62.7 ± 2.6	87.1 ± 6.9

Table 4. Biodistribution data of [^{90}Y]Y-DOTA-Tz in mice bearing PC3 xenografts at 1 h and 2 h p.i. (data expressed as mean %I.D./g and SD, n = 4).

Organs	[^{90}Y]Y-DOTA-Tz	
	1 h	2 h
Blood	1.59 ± 0.62	0.22 ± 0.01
Lung	1.24 ± 0.32	0.31 ± 0.04
Liver	0.76 ± 0.07	0.49 ± 0.15
Spleen	1 ± 0.27	0.33 ± 0.16
Pancreas	0.7 ± 0.32	0.27 ± 0.12
Kidneys	3.65 ± 0.49	2.54 ± 0.71
Salivary Gland	0.68 ± 0.12	0.17 ± 0.02
Intestine	0.41 ± 0.2	0.17 ± 0.05
Stomach	0.65 ± 0.33	0.23 ± 0.08
Bone	1.04 ± 0.25	0.54 ± 0.04
Tumour	1.0 ± 0.4	0.37 ± 0.17
Muscle	0.5 ± 0.2	0.15 ± 0.01

Table 5. Biodistribution data of [^{90}Y]Y-DOTA-Tz-TCO-PEG$_4$-AR in PC3-xenografts bearing mice at 15 min and 1 h p.i. (data expressed as mean % I.D./g and SD, n = 4).

**Organs**	**[^{90}Y]Y-DOTA-Tz-TCO-PEG4-AR**	
	**15 min**	**1h**
**Blood**	14.89 ± 3.89	2.65 ± 0.65
**Thyroid**	6.57 ± 3.95	0.99 ± 0.5
**Heart**	3.55 ± 0.62	0.61 ±0.27
**Lung**	7.48 ± 1.92	1.57 ±0.63
**Liver**	7.35 ± 3.27	0.82 ±0.1
**Spleen**	4.03 ± 2.21	1.08 ±0.39
**Pancreas**	32.02 ± 3.86	14.01 ±1.99
**Kidneys**	20.47 ± 7.97	2.94 ±0.44
**Salivary Gland**	3.69 ± 1.39	0.6 ±0.18
**Small Intestine**	2.74 ± 0.28	0.99 ±0.32
**Large Intestine**	3.98 ± 0.68	0.67 ±0.19
**Prostate**	26.3 ± 18	3.48 ±3.63
**Stomach**	4.72 ± 0.38	1.95 ±0.39
**Bone**	3.15 ± 1.87	0.7 ±0.29
**Tumour**	6.32 ± 1.28	2.26 ±0.93
**Muscle**	2.36 ± 0.33	0.41 ±0.12

Table 6. Comparison of the biodistribution data of [^{111}In]In-DOTA-Tz in PC3-xenografts bearing mice pretargeted with TCO-PEG4-AR (4 h prior administration, 1 nM) with that of preformed [^{111}In]In-DOTA-Tz-TCO-PEG$_4$-AR in the same animal model, at 1 h p.i. (data expressed as mean % I.D./g and SD, n = 4).

**Organs**	***In Vivo* Click reaction**	**[^{111}In]In-DOTA-Tz-TCO-PEG4-AR**
**Blood**	0.28 ± 0.09	1.2 ± 0.4
**Liver**	0.3 ± 0.1	0.74 ± 0.06
**Intestine**	0.12 ± 0.04	1.09 ± 0.08
**Spleen**	0.11 ± 0.04	0.4 ± 0.1
**Heart**	0.12 ± 0.04	0.5 ± 0.3
**Lung**	0.26 ± 0.06	0.7 ± 0.2
**Kidney**	1.5 ± 0.3	3.7 ± 1.1
**Muscle**	0.15 ± 0.11	0.3 ± 0.11
**Bone**	0.21 ± 0.09	0.5 ± 0.1
**Stomach**	0.09 ± 0.06	0.9 ± 0.2
**Tumour**	0.78 ± 0.54	2.5 ± 0.7

14 Figures

Figure SM 1. Representative chromatograms of [^{111}In]In-DOTA obtained by γ-detection (green, front) and In-DOTA-Tz (black, back), obtained by UV-detection.

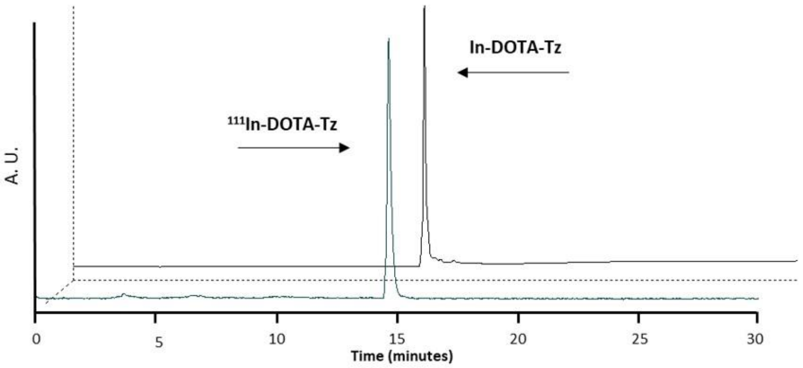

Figure SM 2. HPLC chromatograms of the urine samples from the mice injected with the three radiocomplexes [^{111}In]In-DOTA-Tz, [^{111}In]In-DOTAGA-Tz and [^{111}In]In-DOTA-PEG-Tz.

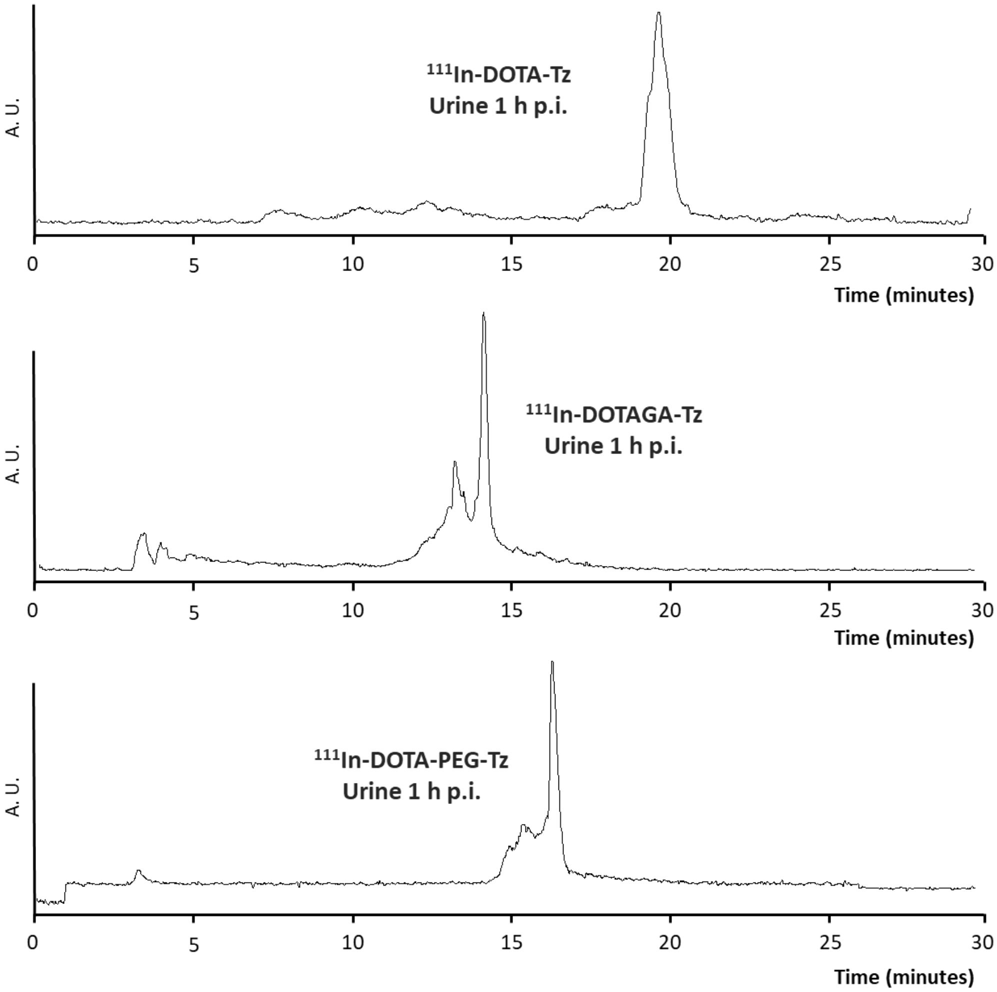

Figure SM 3. Radiochemical purity (estimated by HPLC) of the radiocomplexes [^{111}In]In-DOTA-Tz and [^{111}In]In-DOTAGA-Tz after incubation in different media at 37°C during 24 hours.

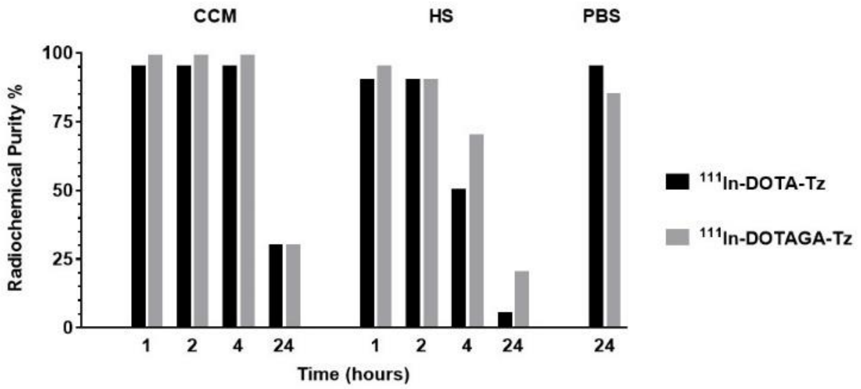

Figure SM 4. HPLC chromatogram (Radiometric System II) of A. [^{90}Y]Y-DOTA-Tz immediately after HPLC purification (R_t = 10.5 min) and B. [^{90}Y]Y-DOTA-Tz two hours after HPLC purification, showing important degradation of the radiocomplex.

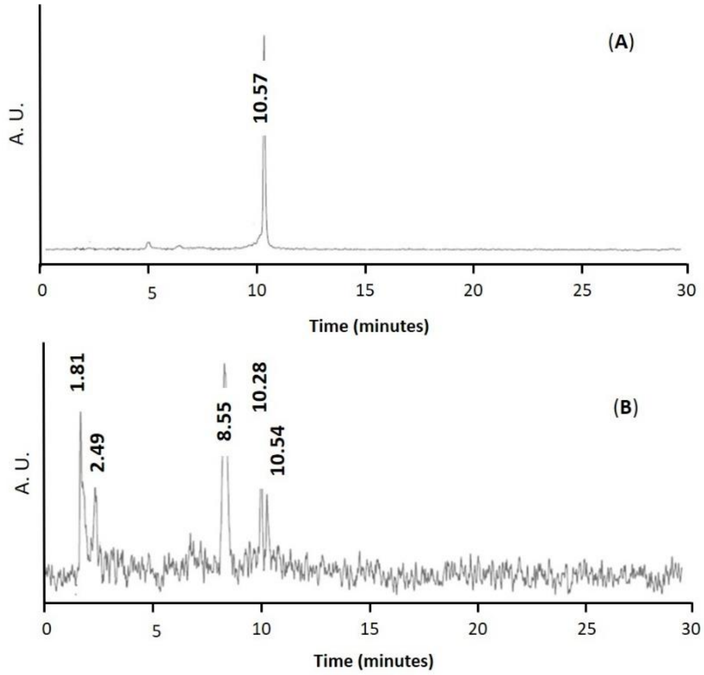

Figure SM 5. HPLC chromatograms (Radiometric System II) of [^{90}Y]Y-DOTA-Tz recovered into 3 µL of ascorbic acid (50 mg/mL) and 147 µL of phosphate buffer 0.4 M, pH 8: (**A**) 1 h, (**B**) 2 h 30 and (**C**) 20 h after radiolabelling.

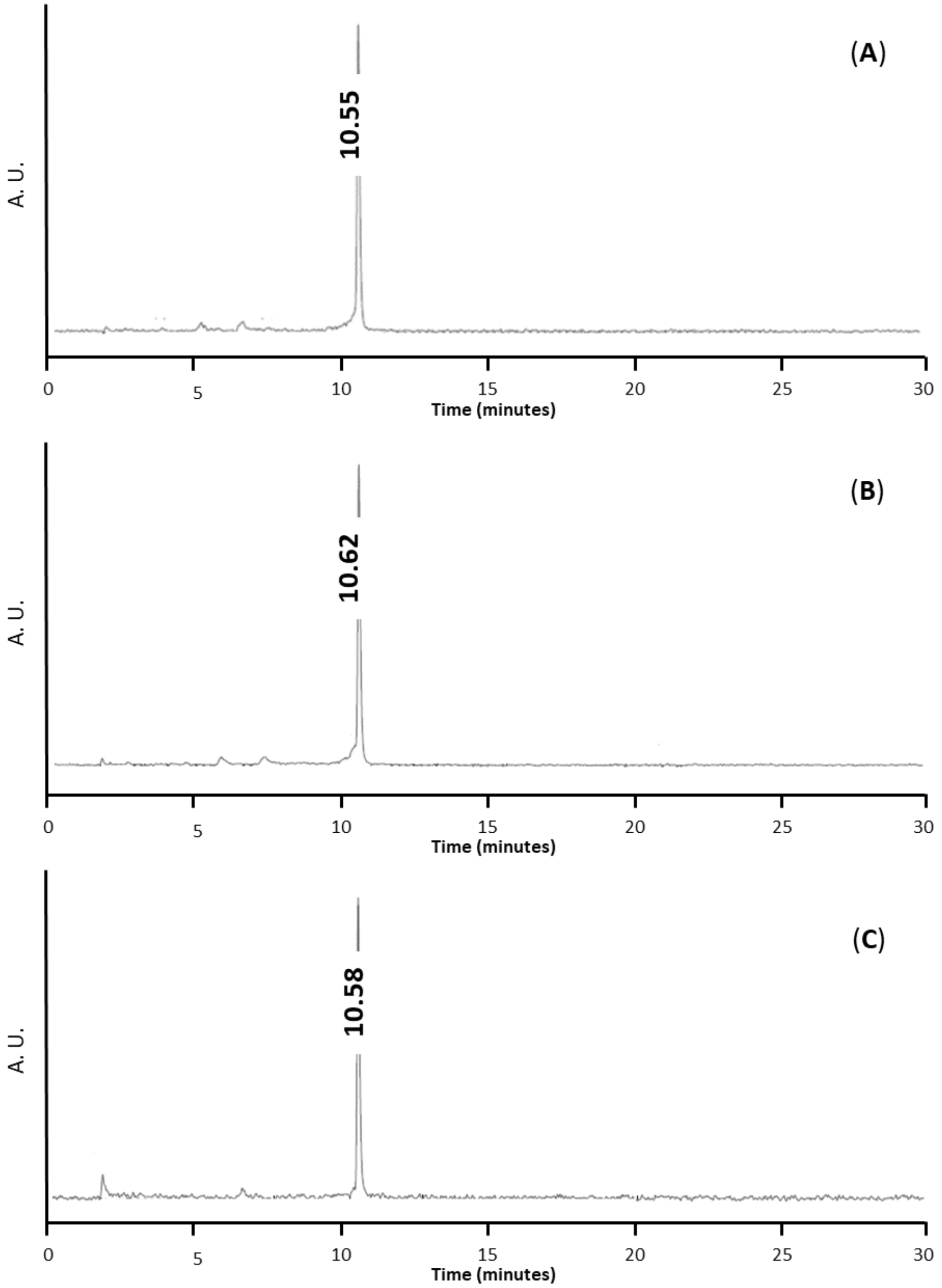

Figure SM 6. Representative HPLC chromatograms (Radiometric System I) of [^{90}Y]Y-DOTA-Tz-TCO-PEG$_4$-AR (R_t = 13.91, γ-detection) right after the radiolabelling.

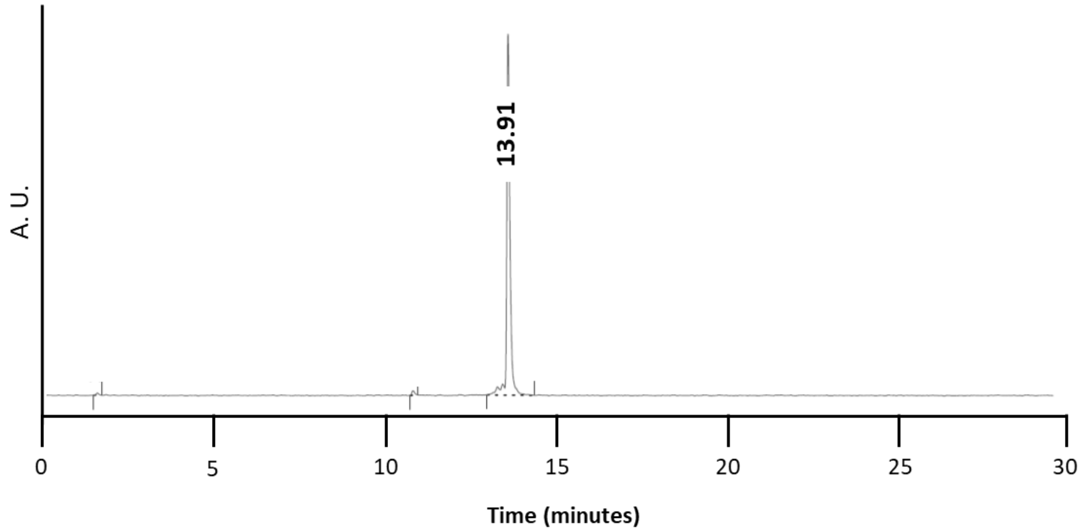

Figure SM 7 A. HPLC chromatogram of the [^{90}Y]Y-DOTA-Tz-TCO-PEG$_4$-AR preparation kept at r.t. during 24 hours (Radiometric System I, R_t = 13.98, γ-detection) **B.** Representative ITLC radiochromatogram obtained after incubation of [^{90}Y]Y-DOTA-Tz-TCO-PEG$_4$-AR (staying at the application point) at 37 °C in human serum (HS) during six days; the elution was done with citrate buffer (pH 5.5, 0.1 M), with the free radiometal ^{90}Y running with the solvent front.

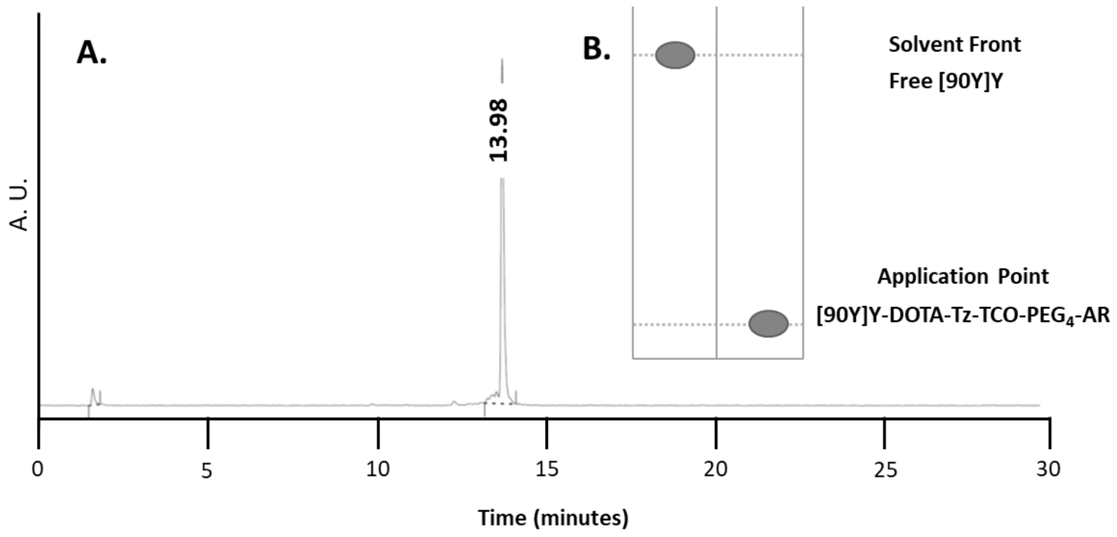

Figure SM 8. HPLC chromatograms (Method 1, γ-detection) of ^{111}In-DOTA-Tz in blue (R_t = 14.8 min) and ^{111}In-DOTA-Tz-TCO-PEG4-AR in black (R_t = 19.2 min).

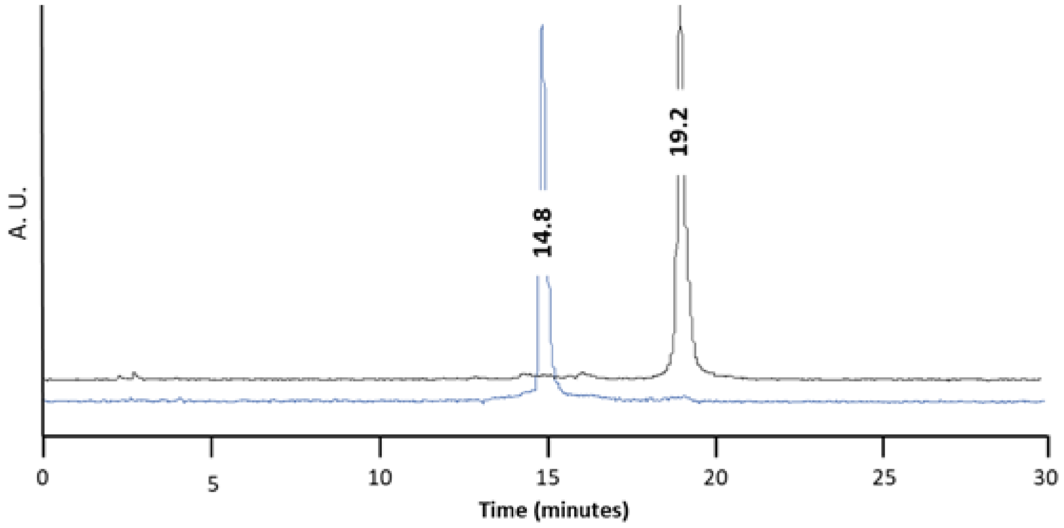

15 References

1. Perrin DD, Armarego WLF, Perrin DR. Purification of laboratory chemicals. Armarego WLF, Perrin DR, editors. Oxford ; New York: Pergamon Press; 1980.

2. Yang J, Karver MR, Li W, Sahu S, Devaraj NK. Metal-Catalyzed One-Pot Synthesis of Tetrazines Directly from Aliphatic Nitriles and Hydrazine. Angew Chemie Int Ed [Internet]. 2012 May 21;51(21):5222–5. Available from: http://doi.wiley.com/10.1002/anie.201201117

3. Wu H, Devaraj NK. Advances in Tetrazine Bioorthogonal Chemistry Driven by the Synthesis of Novel Tetrazines and Dienophiles. Acc Chem Res. 2018;51(5):1249–59.

4. Lang K, Davis L, Wallace S, Mahesh M, Cox DJ, Blackman ML, et al. Genetic encoding of bicyclononynes and trans-cyclooctenes for site-specific protein labeling in vitro and in live mammalian cells via rapid fluorogenic diels-alder reactions. J Am Chem Soc. 2012;134(25):10317–20.

5. Alge DL, Donohue DF, Anseth KS. Facile and efficient Lewis acid catalyzed synthesis of an asymmetric tetrazine useful for bio-orthogonal click chemistry applications. Tetrahedron Lett [Internet]. 2013;54(41):5639–41. Available from: http://dx.doi.org/10.1016/j.tetlet.2013.08.010

6. Hoffman TJ, Volkert WA, Troutner DE, Holmes RA. Reversed-phase HPLC of [99mTc]tetraamine complexes. Int J Appl Radiat Isot [Internet]. 1984;35(3):223–5. Available from: http://www.sciencedirect.com/science/article/pii/0020708X84902400
